# Exploring the impact of a personalised disability reform on people with disability and their primary carers: Evidence from the Australian national disability insurance scheme

**DOI:** 10.1371/journal.pone.0321377

**Published:** 2025-05-07

**Authors:** Bernice Hua Ma, Samia Badji, Gang Chen, Dennis Petrie

**Affiliations:** 1 Centre for Health Economics, Monash University, Caulfield East, Victoria, Australia; 2 Melbourne School of Population and Global Health, University of Melbourne, Parkville, Victoria, Australia; Ankara University: Ankara Universitesi, TÜRKIYE

## Abstract

Australia introduced the National Disability Insurance Scheme (NDIS) in 2013 to provide personalised formal care to individuals under 65 with significant and likely permanent disability. However, many ineligible individuals now face challenges accessing care. Against the backdrop of the introduction of NDIS funding and the simultaneous defunding of other disability services due to the NDIS, this research investigates the short-term impacts of NDIS on the formal service utilisation and carer outcomes for people with profound or severe disability, irrespective of their NDIS status. Using the staggered NDIS rollout, we analyse data from the 2015 and 2018 Survey of Disability, Ageing and Carers. We compare outcomes between primary carers and care recipients in NDIS-available areas (n = 736) and NDIS-not-yet-available areas (n = 318). Results show no short-term impact of NDIS availability on formal service utilisation or frequency, or primary carer outcomes. While some individuals benefit from the NDIS, others may lose access to care. Policymakers should address NDIS equity concerns and consider targeted measures to improve carer outcomes.

## Introduction

There is an international trend towards the personalisation of funding for care services to enable more appropriate and higher quality care. Under this approach, funds are given to people with disability so they can purchase those services that best suit their needs. Australia joined the trend in 2013 by launching the National Disability Insurance Scheme (NDIS) to provide more personalised services to people under 65 with a significant and likely permanent disability. While focusing on supporting people with disability, the NDIS also recognises the essential role of carers and includes them in discussions about support.

**Fig 1:**
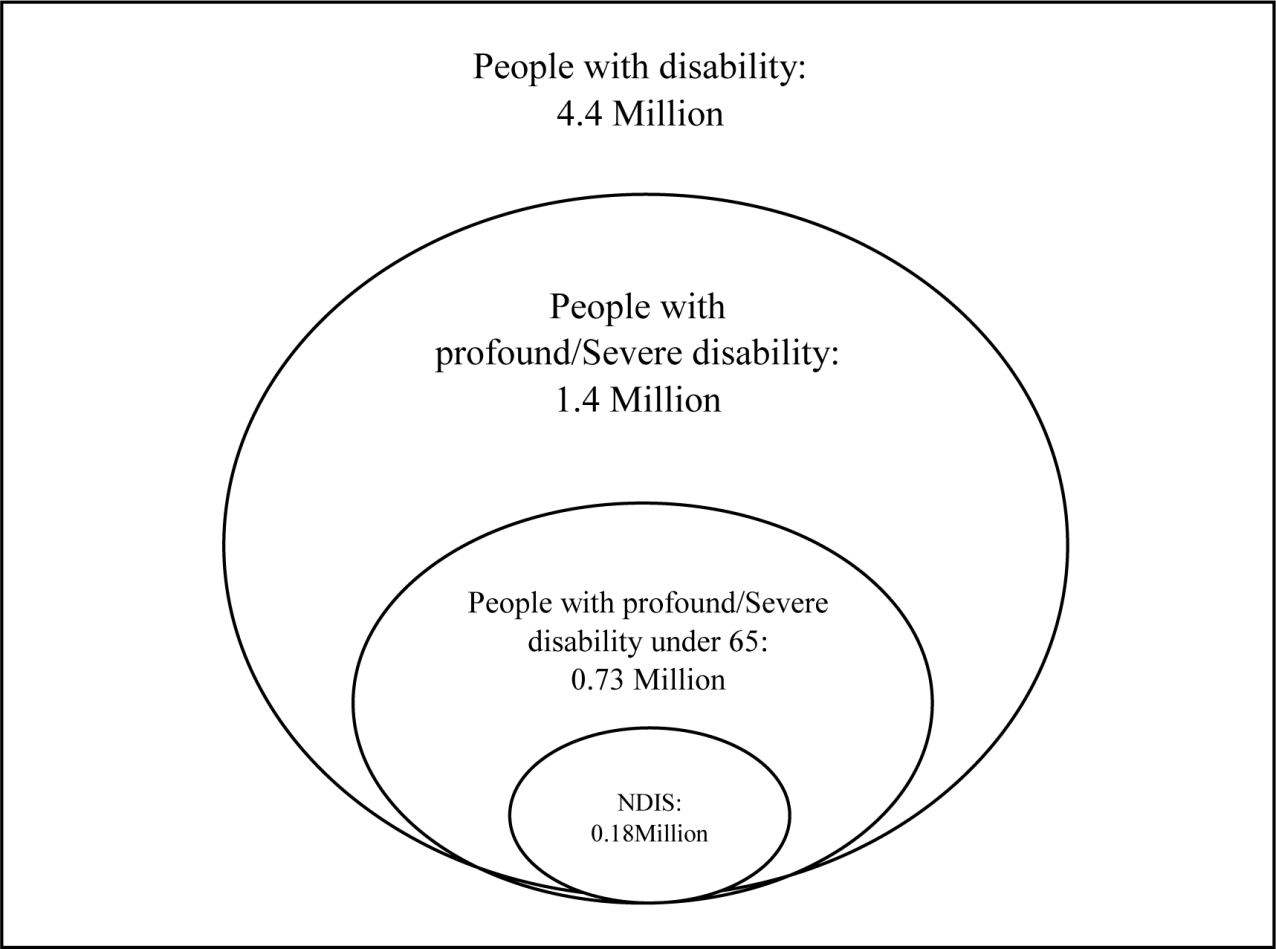
The composition of people with disability in Australia as of August 2018.

The cost of the NDIS is substantial. The projected total Scheme expenses in 2023–24 are AU$41.4, representing 1.6% of the national Gross Domestic Product [1]. The NDIS was particularly targeted at supporting those with high on ongoing care needs. Implementing the NDIS meant not only additional funds to support people with a disability but also a reallocation of public funds away from some national and state or territory-based disability programs that had supported the wider population of people with disability. This included programs like Partners in Recovery, Personal Helpers and Mentors and Day to Day Living [2,3]. Many local governments also ceased providing Home and Community Care programs for adults with disability [4]. As these programs have been scaled back or defunded [5], people not on the NDIS have faced a lack of support, leaving their families as their main source of support. As shown in Fig 1, by August 2018, the NDIS had enrolled a total of 183,965 Australians, which is only one-fourth of the estimated population of people with severe and profound disability under 65 [6,7].

Previous evidence suggested that the NDIS improved outcomes for its participants and carers. For example, participants reported using more formal services to support domestic tasks and seeing specialists more [8]. Their carers also reported taking more frequent breaks [9]. The employment rate also increased for carers of participants under 25 years who had been in the scheme for over a year, with the 15–24-year-old participant group having the most significant improvement [10,11].

The impact of the NDIS on people with severe and profound disability and their carers remains uncertain. This raises significant equity concerns, particularly for those who have not been able to access the scheme. Addressing this evidence gap, this paper estimated whether providing personalised formal services to eligible people with disability in place of access to state-funded organised services for a wider disability group increased formal care use and improved carer outcomes in the short run.

The study uses repeated cross-sectional data (waves 2015 and 2018) of the Survey of Disability, Ageing and Carers (SDAC) [12] to perform an intention-to-treat study. In the first stage, we assessed the changes of the utilisation of formal services for people with disability, as this is a major pathway to change caring hours thus relevant carer outcomes. Then we consider the further downstream impacts on carer outcomes themselves where we also examine the average changes in informal caring hours, employment, and social participation for primary carers. SDAC is useful because it is the only Australian data that was being regularly collected on those with a disability and their carers both prior to and post the NDIS implementation. However, utilising SDAC data to evaluation the NDIS could be challenging especially because of its sample size, which may constrain our ability to interpret the results as being causal. Therefore, we also perform a sample size power calculation to determine what would have been the necessary sample size to detect various possible magnitudes of change.

We first discuss the literature and describe the possible mechanisms through which the NDIS may impact formal service use and carer outcomes. We then lay out the empirical framework, including the data, the identification strategy, and model specification. Finally, we present the results of our analysis and discuss the policy implication.

## Background

### The NDIS

The NDIS provides support to people with permanent and significant disability who enter the scheme before age 65. People with disability usually need their health care provider to prepare evidence stating that their disability is permeant and significant. After gathering the information, they need to submit the evidence to the NDIA. It is ultimately up to the NDIA to decide if an applicant’s disability is permeant and significant and thus meet the eligibility criteria of entering the NDIS. Unlike the previous system, which assumes a one-size-fits-all approach and funds services providers directly, the NDIS considers each individual’s personal goals and preferences, then tailors and provides funds for each individual to spend on the services they need. Three types of support budgets can be funded in one’s NDIS plan: the core supports budget, the capacity building supports budget, and the capital supports budget. The core supports budget, in particular, funds assistance such as self-care activities, household tasks, therapeutic supports not covered by the mainstream health system, and transport if one cannot use public transportation. In addition to the government-funded programs available to support carers, an NDIS participant might use funding in their plan to facilitate respite care, which gives carers short breaks from caring responsibilities. All these services are referred to as “formal services” in this paper, services that are professional, regulated, and typically involve monetary transactions.

There have been several stages of the implementation of the NDIS. It was first tested in trial sites in five states/territories in 2013. Prioritisation was given based on age or residential location. The trial ended in July 2016, and since then, the NDIS began to roll out across Australia (except for some early roll-out areas in NSW). ACT was the first state or territory to complete the roll-out, followed by other states and territories, with Western Australia being the last state to reach the full scheme from July 2023. Details regarding the trial and roll-out dates of the NDIS are listed in Appendix S1Table.

There are several steps to receive a support budget from the NDIS. First, one has to apply for the NDIS with the evidence to prove that they meet the eligibility criteria of entry. Then the National Disability Insurance Agency (NDIA) assesses of the minimum eligibility requirements, and will notify the applicant if they are granted access to the NDIS. Planning meetings between an NDIA planner and the participant will take place if access is granted to discuss how much support the participant is likely to need and a plan will be made accordingly. The NDIA then reviews the plan. Once the plan is signed off by the NDIA, participants can arrange their services [13]. The NDIS participants could choose whether their NDIS plan is managed by themselves, by the NDIA or by a plan manager. About 70% of people with disability choose to use a plan manager who helps them with reimbursement and fund monitoring but does not support coordination [14]. About 40% of NDIS participants also have support coordination funded, which helps them coordinate support and services; however, research showed that only about 20% of the participants were able to use at least 80% of their support coordination funding [15]. For those NDIS eligible, the waiting time to get an approved plan could be long, especially during the early stages of the NDIS roll-out. It was estimated that it took more than seven months for a participant to get an approved plan before July 2019 [11], not to mention the time needed to arrange services.

### Mechanisms

**Fig 2:**
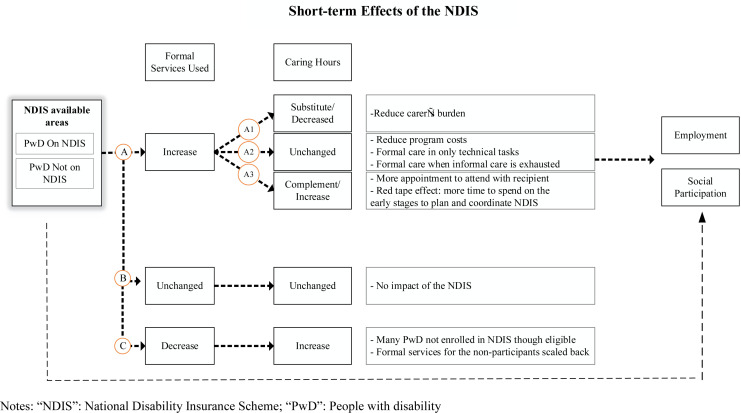
The potential mechanism through which NDIS may affect caring hours, employment and social participation of primary carers.

Fig 2 illustrates the potential mechanisms through which the NDIS roll-out may impact carer outcomes through its impact on formal service utilisation, as the latter is a core funding area of the NDIS. Our study examined the average effect of the NDIS by comparing two key groups – people with profound or severe disability living in areas where the NDIS has been rolled out, and those living in areas where the NDIS has not been rolled out. Given that formal services are a core area funded by NDIS, eligible people living in areas where the NDIS is available could receive more and higher quality formal services and, consequently, the average formal service use in the NDIS available areas may increase (Path A, Fig 2). The literature has competing views on whether increased formal caring hours substitute, complement or are independent of informal caring hours. According to the substitution theory, an increased supply of formal care may decrease informal care [16–20] (Path A1, Fig 2). However, if formal care is provided for tasks that informal carers cannot perform [21] or if it is provided only when the informal care has been exhausted [22], the informal care may remain unchanged even if the formal care increased (Path A2, Fig 2). As highlighted in the NDIS guidelines, the NDIS was not intended to replace informal care; in such circumstances, formal care and informal care may be independent. It is also possible that NDIS increased the informal caring hours as the complementary theory postulates (Path A3, Fig 2). There is not much evidence about the reinforcing effects of formal care. However, more informal care may be needed to permit access to the formal care being provided (e.g., if one needs help to undertake formal care activities). Also, at the early stages of the NDIS roll-out, carers may have needed to spend more time planning and coordinating the approved services for the recipients; therefore, their caring time may have increased.

Those ruled ineligible for the NDIS but who may have previously accessed state/territory-based services may now face difficulties in accessing formal services. As a result, the average use of formal services in the area may not change (Path B, Fig 2) or decrease (Path C, Fig 2) even if the NDIS has become available. The caring hours are only expected to change if there is a change in the use of formal services. However, if the formal service use decreases, the caring hours may need to increase to fill this gap.

Given the changes in caring hours, the NDIS may or may not affect labour force and social participation outcomes through the change of caring hours (Path A1 and A3, Fig 2). It is possible that the decreased informal caring hours free up some of the informal carer’s time so that informal carers can work or participate in social events because previous research found that caregiving is associated with higher rates of part-time work and fewer hours of work [23,24]. However, if the NDIS increased informal caring hours, the carers may be less likely to be employed or enjoy the same leisure time simply because they have no free time. There is also a possibility that the NDIS influences employment and social participation outcomes through other channels that are independent of the caring hours. For example, family support and counselling may help the family of people with a disability to manage the demands the disability brings. If mental pressure prevents carers from participating in economic or social activities, then the NDIS may indirectly support carers in this way as well.

This study examines changes occurring within a relatively brief period of 2.5 years maximum ---- a duration considerably shorter compared to the 10-year milestone reached by the NDIS. The impact of the NDIS may be different in the short term compared to the long term. While the impact on caring hours and social participation may show relatively quickly after getting formal care, the effects on employment may take longer to manifest. Also, the impact on employment may depend on the flexibility of available jobs — for example, the number of part-time jobs in the labour market.

## Methods

This study estimates the impact of the NDIS on the use of formal services of people with disability who have a profound or severe disability (or needing aids) under the age of 65, as well as the caring hours, employment and social participation of their primary carers before and after the NDIS has become available in their areas. To minimise bias from factors other than the intervention, we compare people with profound or severe disability (or needing aids) under the age of 65 who lived in areas where the NDIS are available to a similar control group but who were living in areas where the NDIS was not yet available to them. We also control for the characteristics of the primary carer and recipient dyad and area-level fixed effects. The analysis is run on a repeated Australian cross-sectional survey—Survey of Disability, Ageing and Carers (SDAC), the most detailed and comprehensive source for examining people with disability and their carers in Australia.

### Data

#### Household level dataset: survey of disability, ageing and carers (SDAC).

This study uses mainly the cross-sectional data from SDAC waves 2015 and 2018 which each include approximately 20,000 responding households. These are the only SDAC waves that contain information about an individual’s local government area (LGA)—the third level of government in Australia at the time of analysis.

#### Self-constructed data on NDIS availability at the LGA level.

We use the LGA to determine if the individual is in an area where the NDIS has become available. The NDIS availability date for each LGA is extracted from the agreements the states and territories signed with the Commonwealth government.

#### Local unemployment rate.

We also control for the local unemployment rate in our analysis and use data from the Australian National Skills Commission to extract this information (National skills commission, 2022).

Ethics was approved by the Monash University Human Research Ethics Committee (MUHREC) (Project ID: 21758). The authors gained access to the data on 10th May, 2020.

### Sample

#### Identification of the primary carers and recipient(s) dyad.

We match each primary carer to their care recipient. Some primary carers in SDAC cared for more than one recipient, and some recipients had multiple primary carers. In this situation, we kept only one primary carer and recipient pair, prioritising those identified as in a primary carer and main recipient relationship (85%), followed by the primary carer and non-main recipient relationship. We also restricted the sample to those recipients potentially eligible for the NDIS who have a profound or severe disability or need an aid and are under the age of 65. Our final sample comprises 1,054 primary carers of recipients with profound or severe disability (or needing aids) under 65. Further details of the sample size can be found in the Appendix S1Fig flowchart.

#### Treatment and control groups.

The NDIS trials and roll-out targeted different population groups. Some states or territories prioritised certain ages, while some were geographical.

Both the treatment and control groups consist of people with profound and severe disability or who need an aid/s who meet the age criteria and their primary carers. The primary carers are aged over 14 years and live in the same private dwelling with their recipient of care. The treatment group are people who lived in the LGAs where the NDIS became available between the two waves of SDAC interviews, i.e., 1 January 2016 and 30 June 2018 (“*the treatment period*”). In the control group, people lived in the LGAs where the NDIS became available only after the 2018 SDAC interview, i.e., after 1 July 2018 (“*the control period*”). We exclude people living in the LGAs that received the NDIS before 31 December 2015 as we do not have relevant data before 2015 (i.e., they were always treated). We also excluded people from Western Australia (WA) because WA ran its own trial, which extended into our analysis period, and the NDIS delivery model in the trial differed from the other states and territories.

**Fig 3:**
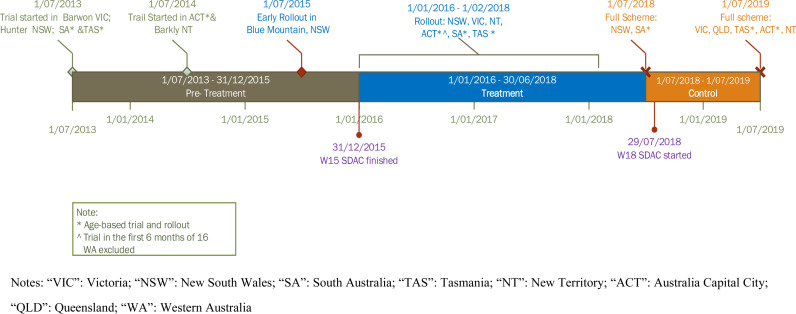
NDIS Implementation Timeline and Research Design.

Details of the timeline are presented in Fig 3, and the details of the trial and roll-out criteria can be found in Appendix S2 Table. Characteristics of the treatment and control groups are presented in the following subsection.

### Outcome of interest

As the carer’s outcomes can be changed through the NDIS’ impact on formal services utilisation of the care recipient, this paper focuses on two sets of outcomes: the first set encompasses formal service use by the care recipients and the second set, the outcomes of the primary carers.

For the first set of outcomes at the care recipient level, we use questions about the frequency of assistance received from organised services because of their disability. Health care services are excluded as we cannot distinguish from the survey which ones are covered by the health system (Medicare) versus funded by other means. The survey participants were asked: “On average, how many times per [day/week/month/year]?” if they stated they needed organised care. The frequencies are transformed into ‘per week’ amounts if the original answer was provided in times per day/month/year assuming there are 52 weeks in a year. For example, if the answer is three times per month. Then we assume that it is 36 times (12months *3) per year. This is then converted to 0.69 = 36/52 times/week. We consider the overall numbers of formal services along with the extensive margin (i.e., whether they used any formal services) and the intensive margin (i.e., how many formal services they used when they had used at least some).

In the second set of outcomes at the carer level, our main outcome of interest is the weekly caring hours of primary carers for recipients. We also explore employment and social participation as those will likely change through the change in caring hours. We focus on co-inhabiting primary carers because they tend to care for longer hours, and the NDIS may have the greatest impact on them. Primary carers consist of 35% of all carers in our data, and they were asked: “On average, how many hours do you spend each week providing care to (recipient X)” This variable is converted into a continuous variable from a 6-level categorical variable (<9, 10–19, 20–29, 30–39, 40–69, 60 + hours) using mid-point transformation.

We also analyse the impact on employment and social participation of the primary carers. Our employment indicator is equal to one if the respondent reported they were employed full-time or part-time (and zero if they were unemployed, looking for work or not in the labour force). For social participation, we look at both social participation without the recipient (one if the primary carer had been to certain places or events in the last 12 months without the recipient and zero otherwise) and social participation with or without the recipient.

Further details of how the variables are constructed are listed in Appendix S3Table.

### Covariates

We control for several covariates likely to be associated with our outcome of interest but unrelated to NDIS access to increase our estimation precision. The covariates include two sets. The set of carer characteristics includes age, sex, education level and the number of recipients of care. The second set is recipient-level characteristics and includes age, sex, marital status, education level, whether born in Australia, disability severity levels, disability types, the Accessibility Remoteness Index of Australia (ARIA) to reflect the ease or difficulty people face accessing services, the number of adults (aged 15 or more) without disability living in the household, and the number of bedrooms in the household. The last one is regarded as a proxy for the income level of the household, as the income variable has 20% of missing values. Unemployment rates by LGA one year before the start of each wave of survey (September 2014 to June 2015; September 2017 to June 2018) are also controlled for to account for local labour market conditions.

### Estimation strategy

To start with, we compare the impact of the NDIS on formal service use of people with profound or severe disability (or needing aids) under 65 living in areas where the NDIS became available between January 2015 to June 2018 and the caring hours, employment and social participation for their primary carers to those with the same characteristics but living where the NDIS was not available until after July 2018 (i.e., after when the 2018 SDAC interview took place). Between the first quarter of 2015 and the second quarter of 2018, the NDIS covers approximately one-fourth of the total population of individuals with severe and profound disabilities in those specific areas. Given that our data does not include information on actual NDIS enrolment, the results represent intent-to-treat (ITT) estimates. ITT estimates are informative for understanding the population-level impact of the policy change. In our case, the results show the change of outcome attributed to the NDIS roll-out (and withdrawal of other services) on all the people with profound or severe disability or needing an aid (aids) who meet the age criteria in the NDIS available areas.

Our identification of the NDIS impact relies on LGA variation in the timing when the NDIS becomes available. Therefore, we need to ensure that the impact of the NDIS on our outcomes is not attributed to other LGA-level factors associated with the availability of the NDIS. We use LGA fixed effects to control for the potential correlation between the NDIS roll-out and time-invariant unobservable LGA characteristics. Simple pre- and post-treatment comparisons could also be influenced by the time trends in the outcome variables and other national policy changes or events; therefore, we then estimate the effect by applying the difference-in-difference (DiD) method using OLS regression. Our specification is as follows:


$$y_{ijlt}\;=\;\beta_{0}+\beta_{1}(Post_{t}\;*\;T_{il}+\;\lambda^{\prime }Z_{ijt}\;+\;v_{t}\;+\alpha_{l}\;+\epsilon_{ijlt}$$ (1)

For the formal service outcomes, y represents the outcome for the overall number of formal services used, the intensive and extensive margin of the formal services of recipient *j* living in LGA 𝑙 during wave 𝑙. For the carer outcomes, the dependent variable *y* corresponds to outcomes of carer *i* of recipient *j* living in LGA 𝑙; during wave 𝑙. The outcomes include weekly caring hours, employment and social participation. 𝑙𝑙𝑙𝑙_𝑙_ equals one if the outcome is measured in 2018 and zero otherwise. *T*_il_ equals one if people are in the treatment group and zero if they are in the control group.

The parameter of interest is 𝑙_1_, representing the impact of the NDIS on the treatment group relative to the control group after the NDIS became available. This assumes that the differences in the outcomes are attributed to the random-like roll-out of the NDIS participation at the LGA level, not other factors.

We also control for the characteristics of the recipients 𝑙 and the primary carers 𝑙 and the unemployment rate, which are included in vector *Z*_𝑙𝑙𝑙_. Time-fixed effects 𝑙_𝑙_ control for common nationwide policy changes such as changes in welfare payments, the availability of new medicines and other nationwide trends between the two waves. The LGA fixed effect 𝑙_𝑙;_ controls for any time-invariant differences across LGAs that may be correlated with our outcomes, such as demographic composition, supplier market, disability coordination facilities, cultural values, or economic conditions which are crucial factors that may impact our outcomes. 𝑙_𝑙𝑙𝑙𝑙_ is the error term. We do not further match our sample based on characteristics such as age and gender within each LGA as suggested in other literature [25] because there are, on average, only 5 observations per LGA.

The previous evaluation reports on NDIS found different effects for different age groups, and positive employment changes were observed in the carers of participants aged 15–24 [10]. Therefore, we also perform a subgroup analysis for recipients aged 15–24 versus those aged 0–14 and 25–64, who are within the NDIS eligible age criteria.

A key assumption in DiD analysis is that in the absence of the treatment, the trends in the outcomes between the treatment and the control groups would have been the same (the parallel trend assumption). In our study, we cannot test this assumption, given that only two waves of data are available. In the following section, we examine the robustness of the results to a different control group—primary carers of recipients who are not eligible for the NDIS (e.g., the disability status of the recipients is not profound or severe, or they are aged 65 years and older) and they live in same LGAs where the NDIS has not been rolled out.

Given that repeated cross-sectional data is used, we need to check that the time-invariant characteristics of the participants in our sample did not change dramatically between the two waves to perform a valid DiD analysis. Two-sided T-tests and Chi-square tests are performed to compare the characteristics of the primary carers and recipients in our treatment and control group between the waves.

We perform multiple robustness tests for our carer outcomes, including sensitivity to the region-level fixed effects, cut-off dates and different sample restrictions. In order to minimise the impact of different characteristics of the sample on the results, in one of the robustness checks, we use propensity score weighting to weight the entire sample from Wave 15 and the control group from Wave 18 against the treatment group of Wave 18 for characteristics including age, sex of the carers, and the sex, disability status, disability type, marital status, education level, whether born in Australia, number of bedrooms, number of adults without disability in the household, and the rurality (Accessibility/Remoteness Index of Australia or ARIA) of the recipients of care’s residence. We bootstrapped the sample 1,000 times to account for the uncertainty around the weights and the estimates of the weighted sample. We also interacted individual characteristics with the treatment variable (𝑙𝑙𝑙𝑙_𝑙_ #*T*_𝑙𝑙_) and estimated the average effect to account for potential heterogenous treatment effects.

We also ran a placebo analysis with primary carers of people with disability who are not eligible for the NDIS because they are older than age 65 or who have mild disability using our treated and control group LGA. This estimates whether there is any effect for those not targeted by the NDIS.

### Minimum sample size estimation

Given that the SDAC survey was the most comprehensive source of information available to understand different effects of the NDIS, we wanted to understand how big the SDAC sample frame needed to be in order to detect economically meaningful effect of the NDIS.

We therefore hypothesized several scenarios of different impact sizes and computed the minimum cluster size needed to detect these potential impacts.

SDAC’s sampling is clustered at the LGA level. We therefore computed cluster size conditional on the maximum number of LGA possible. There were 407 LGAs in Australia in 2018 - excluding the state of Western Australia which had its own NDIS trial at different periods. We also excluded three LGAs in other states because they were also the early trial sites (Hunter NSW, Barwon VIC and Barkly NT) and therefore 404 LGAs remained.

We assumed an equal number of LGAs in the control and treatment groups to compute the minimum number of people per LGA under different assumptions of intraclass correlations that would be needed to detect meaningful changes in outcomes. We used the means and standard deviations of the control group in the post treatment period, as a reference point for all computations - last column in Table 1. All results are for an 80% power test using a 5% two-sided test.

**Table 1:** Descriptive Statistics (N = 1,054).

Mean (SD)/ N (%)	Treatment 15 (n = 439) (1)	Control 15 (n = 182) (2)	Treatment 18 (n = 297) (3)	Control 18 (n = 136) (4)
**Outcomes (For primary carers)**				
Total formal services (times/week)	5.74 (16.31)	6.27 (17.51)	5.33 (18.05)	4.69 (16.29)
Weekly caring hours	32.75 (22.54)	34.67 (23.17)	32.62 (22.45)	29.68 (23.50)
Employed (FT or PT) ^	0.47 (0.50)	0.49 (0.50)	0.52 (0.50)	0.57 (0.50)
Social participation without recipient	0.55 (0.50)	0.52 (0.50)	0.60 (0.49)	0.61 (0.49)
Any social participation	0.78 (0.41)	0.74 (0.44)	0.76 (0.42)	0.80 (0.40)
**Carer Characteristics**				
Age	48.11 (13.57)	46.41 (12.57)	50.15 (12.87)	48.05 (11.97)
Male	0.34 (0.47)	0.32 (0.47)	0.30 (0.46)	0.31 (0.47)
Number of recipients of care	1.42 (0.74)	1.29 (0.54)	1.38 (0.66)	1.40 (0.70)
Adults (>=15) without disability in household	1.07 (1.00)	1.25 (1.02)	1.22 (0.97)	1.32 (1.12)
Highest education level [N (%)]				
*Year 11 and below*	148 (0.34)	58 (0.32)	73 (0.25)	37 (0.27)
*Year 12*	79 (0.18)	35 (0.19)	73 (0.25)	32 (0.24)
*Certificates/diploma*	161 (0.37)	69 (0.38)	119 (0.40)	54 (0.40)
*Bachelor and above*	51 (0.12)	20 (0.11)	32 (0.11)	13 (0.10)
**Recipient Characteristics**				
Age	36.51 (21.15)	34.71 (21.15)	37.68 (21.62)	36.48 (21.66)
Male	0.52 (0.50)	0.54 (0.50)	0.52 (0.50)	0.64 (0.48)
Married/ De facto	0.47 (0.50)	0.43 (0.50)	0.49 (0.50)	0.51 (0.50)
Disability status (1. Profound; 0. Severe)	0.95 (0.22)	0.97 (0.16)	0.95 (0.22)	0.96 (0.19)
Born in Australia	0.83 (0.37)	0.83 (0.38)	0.81 (0.39)	0.78 (0.41)
Number of bedrooms in household	3.26 (0.86)	3.36 (0.87)	3.33 (0.90)	3.52 (0.77)
Highest education level [N (%)]				
*Year 11 and below*	266 (0.61)	105 (0.58)	170 (0.57)	72 (0.53)
*Bachelor and above*	32 (0.07)	18 (0.10)	26 (0.09)	12 (0.09)
*Certificates/diploma*	93 (0.21)	35 (0.19)	66 (0.22)	37 (0.27)
*Year 12*	48 (0.11)	24 (0.13)	35 (0.12)	15 (0.11)
Rurality [N (%)]				
*Major cities*	262 (59.68)	122 (67.03)	176 (59.26)	100 (73.53)
*Inner regional*	120 (27.33)	16 (8.79)	90 (30.30)	23 (16.91)
*Outer regional and remote*	57 (12.98)	44 (24.18)	31 (10.43)	13 (9.56)
Disability Type [N (%)]				
*Psychosocial*	72 (0.16)	31 (0.17)	46 (0.15)	23 (0.17)
*Other*	367 (0.84)	151 (0.83)	251 (0.85)	113 (0.83)

## Results

### Descriptive statistics

Table 1 presents the summary statistics of the sample by the NDIS participation group and wave, and Appendix S4 Table shows the results of our comparison tests. Of the 1,054 primary carers and recipient dyads, 439 are in the treatment group observed in wave 2015, 297 are observed in wave 2018, 182 are in the control group observed in wave 2015, and 136 are observed in wave 2018. The proportion of male recipients is higher in the control group observed in 2018 than in the other groups. There are differences in the number of bedrooms among the groups, although the effect size is small (max 0.3 rooms). People are more likely to live in major cities if they are in the treatment group. The average age of the primary carer was two years older for both the treatment and the control groups in 2015 than in 2018. There are fewer adults without disability in the household in the treatment group observed in 2015 than in other groups.

### Main results

The estimated impact of the NDIS roll-out on the primary carers’ outcomes and the formal service used by people with disability are shown in Table 2. The coefficient for the variable *NDIS available area# Wave18* reflects the absolute additional change in the outcomes between 2015 and 2018 for those who live in the LGAs where the NDIS had been rolled out between 2015 and 2018 (the treatment group) versus where the NDIS had not yet been rolled out (the control group). The outcomes include (1) overall number of formal services, (2) the extensive margin of formal services, (3) the intensive margin of the formal services, (4) weekly caring hours, (5) employment status, (6) social participation without recipients, (7) social participation with or without recipients. The complete list of covariates and results are provided in Appendix S5 Table.

**Table 2:** The association between NDIS availability in an LGA and changes in the outcomes of interest.

	(1) Formal services overall (times/week)	(2) Formal services extensive margin	(3) Formal services intensive margin (times/week)	(4) Caring hours (hours/week)	(5) Employment	(6) Social participation (Alone)	(7) Social participation (Any)

**NDIS available area# Wave18**	0.073	-0.102	-0.719	4.787	-0.053	-0.067	-0.097
	(2.494)	(0.0739)	(4.315)	(3.853)	(0.081)	(0.079)	(0.063)
*N*	1,052	1,052	511	1,052	939	1,052	1,052
R-squared	0.154	0.134	0.228	0.313	0.166	0.108	0.118
Number of LGA	205	205	160	205	194	205	205

**Notes:** “NDIS available areas # Wave 18”: The impact of the NDIS on the treatment group relative to the control group after the NDIS became available. All estimations are conducted using OLS regression. Outcomes of (2), (5)-(7) are binary outcomes, while (1), (3) and (4) are continuous. The regressions control for carer characteristics, including age, age square, the number of recipients of care, sex, highest education levels, adults (>15yo) without disability in the household; the recipient characteristics, including age and age square, sex, marital status, highest education level, disability status, disability type, rurality, and the number of bedrooms in the household. They also control for the LGA-level fixed effect, the time-fixed effect and the unemployment rates. Robust standard errors are in parentheses, and they are clustered on the LGA level. The key variable (interaction term) of interest in this table is insignificant (P > 0.10).

We did not detect statistically significant NDIS impacts on any of the outcomes. The results show that the people with a profound or severe disability (or needing aids) who live in areas where the NDIS became available are, on average, nearly 10% less likely to use formal services compared to their counterparts in areas where the NDIS had not yet been implemented after the reform; however, the result is insignificant. For those who use formal services at least once per year, the NDIS reform did not lead to a statistically significant difference in the frequency of formal service use between the treatment and the control group.

For the carer outcomes, the weekly caring hours of primary carers increased by 4.8 hours between 2015 and 2018 for the carers in the NDIS area group relative to the control group, though it was not statistically significant. We find that primary carers in the treatment group are five percentage points less likely to have part-time or full-time work after the NDIS roll-out (β= -0.053, SE = 0.081) compared to the control group, but this result is not statistically significant. We find no statistically significant impact on participation in any social activity (β = -0.067, SE = 0.079) or participation in social activity without the recipient (β = -0.097, SE = 0.063). We then explore if the decrease in social participation is driven by the change in the social participation of recipients of care. This may reduce the chance of the primary carer accompanying the recipient to those events. The results (Appendix S6 Table) do not show a statistically significant effect (β = 0.101, SE = 0.115).

While previous reports have suggested possible gains for carers of young people, our subgroup analysis shows no significant heterogeneity in results for the 15-to-24-year-old age group (Appendix S7 Table).

### Robustness checks and placebo test

We conduct multiple analyses to explore the sensitivity of our results on the carer outcomes to various levels of fixed effects, cut-off dates, sample restrictions and sample weightings. A summary of the robustness test results is presented in Table 3, and fully separated results are shown in the appendix. Generally speaking, all the results consistently show no significant effects. Although some of the results changed sign, as they stay statistically insignificant, it could be driven by random variation rather than meaningful effects because of the limitation of small sample size. First, due to the concern that the characteristics of our 2018 sample may differ from the characteristics of our 2015 sample, we weighted our sample based on the characteristics of the NDIS participants in the 2018 sample as described in Section 3.6 and bootstrapped the sample 1,000 times. The results are similar to our main analysis using the unweighted sample. We do not find statistically significant changes across the seven outcomes. (Appendix S8 Table). We then explore if the fixed effect at the regional level instead of the LGA level would impact the statistical power of our analysis. Since the NDIS roll-out is based on the regional level information, then broken down to the LGA level in our main analysis, the treatment and control groups remain identical. With the regional fixed effects, we find similar trends with a negative coefficient for employment and social participation and an increase in caring hours. All the results are statistically insignificant (Appendix S9 Table).

**Table 3:** Robustness checks and placebo test: estimated association between NDIS availability and outcomes under *alternative* assumptions.

	(1) Formal services overall (times/week)	(2) Formal services extensive margin	(3) Formal services intensive margin (times/week)	(4) Caring hours (hours/week)	(5) Employment	(6) Social participation (Alone)	(7) Social participation (Any)
Main analysis	0.073	-0.102	-0.719	4.787	-0.053	-0.067	-0.097
Weighted sample	-0.469	-0.157	0.260	1.977	-0.022	-0.012	-0.060
Regional fixed effects	0.989	-0.096	5.243	5.673	-0.086	-0.066	-0.100
Cut-off 17	0.352	-0.029	-3.910	1.894	-0.088	-0.033	-0.135*
Main recipient	-0.756	-0.074	-4.833	4.804	-0.111	-0.108	-0.115*
Excl. eligible carer	-0.295	-0.094	-0.975	4.295	-0.047	-0.075	-0.101
Diff control group	0.165	-0.069	2.345	1.045	0.070	0.067	0.012
#Characteristics	0.333	-0.107	-0.322	4.333	-0.053	-0.064	-0.100
Placebo	0.466	0.466	-0.152	-5.438	0.078	-0.021	-0.055

**Notes: Each cell corresponds to a different regression of the outcome variables of interest (in column) on the interaction term of interest and/or subsample of interest as identified by the row.** All the robustness checks and placebo tests are performed using Difference in Difference with LGA fixed effects, except for “Regional fixed effects”, where the fixed effects are at the regional level instead. The robustness checks are conducted using different samples to test different assumptions as stated in the main text.

**“Main sample”** repeats the results presented in Table 2 for comparison. **“Weighted sample”**: We weighted characteristics of the wave 18 sample based on the wave 15 sample (main analysis: unweighted sample); **“Regional fixed effect”:** A region is consisted of several LGAs. The robustness check was using DiD with Regional Fixed effect (main analysis: LGA fixed effects); **“Cut-off 17”:** The treatment group is composed of people who lived in areas where the NDIS became available between January 2016 and 30 June 2017 (main analysis: sample between 1 January 2016 and 30 June 2018); **“Main recipient”:** Restricted the sample to the primary care and main recipient relationship (main analysis: primary carer and any caring recipient); **“Excl. eligible carer”:** Excluded primary carers who also have profound or severe disability under 65 and are living in NDIS available areas between the two waves of surveys (main analysis: including carer who may be eligible for the NDIS); **“Diff control group”:** Changed the control group to primary carers of recipients who are unlikely to be eligible for the NDIS (because of age and disability severity level) and live in the same areas as the treatment group in our main analysis (areas with NDIS) (main analysis: people with disability living in LGAs which NDIS was available late); **“#Characteristics”:** We added interaction terms between the main effect and the carer and care recipients’ characteristics (main analysis: no interaction terms); “Placebo”: Treatment group is consisted of people with mild or moderate disability or older than 65 who are not eligible for the NDIS (main analysis: Severe or profound disability under 65 who are likely to be eligible for the NDIS).

*p < 0.10; we also tested for p < 0.01, < 0.05, and the results were statistically insignificant on these levels.

The primary carer may suffer short-term negative impact when the NDIS is being coordinated. Therefore, we also explore if the results differ for those whose areas that had NDIS available for at least one year. We vary the cut-off date for those considered to be treated from 30 June 2018–30 June 2017 when constructing *T*_𝑙𝑙_, and keep the same control group to ensure that the differences in the results are not due to the change in the control groups. The results in Appendix S10 show that people living in areas where NDIS was available before June 2017 were 14% less likely to participate in social activities with or without the recipients of care, and the result is significant at the 10% level (β = -0.135, SE = 0.071) (Appendix S10 Table)

Primary carers may spend more time caring for the main recipients, and therefore, the effect size may become larger if the sample is restricted to main recipients only. We restrict our sample to the primary carer and main recipient relationship by excluding the non-main recipients in our sample (7.0%). The results are similar to our main analysis except that the possibility of participating in social activities with or without the recipients becomes significant at the 10% level (β = -0.115, SE = 0.065) (Appendix S11 Table).

About 10% of our primary carers in the sample are themselves eligible for NDIS and live in LGAs where the NDIS was available between 2015 and 2018. Therefore, there is a concern about whether the carer’s potential participation in the NDIS would also impact caring hours. We ran a sensitivity analysis restricting our sample to carers not eligible for the NDIS or living in LGAs where the NDIS is unavailable during the treatment period. Our main results were robust to this approach (Appendix S12 Table).

We also examine the robustness of the results with a different control group—primary carers of recipients who are unlikely to be eligible for the NDIS (because of age and disability severity level) and live in the same areas as the treatment group in our main analysis (areas with NDIS). This was done because we could not test for the parallel trend assumption, and we expected the results to be similar because the NDIS should also not affect this alternative control group. All the results remain statistically insignificant, although the outcomes of employment, social activities, and the frequency of using formal services are slightly above zero (Appendix Table S13).

Lastly, we examine the heterogeneous treatment effects by interacting the carer and recipient characteristics with our treatment term. We find no significant change to our main results (Appendix Table S14)

The results of the placebo analysis with the population of people with mild or moderate disability or older than 65 show no statistical changes in the outcomes. It indicates that the NDIS reform has no or minimal effects on the population, which is not targeted, consistent with our expectations (Appendix Table S15).

### Minimum sample size estimation

We wanted to understand for different possible impacts, how big SDAC would have needed to be to detect meaning changes in the outcomes. The sample means in Appendix Figs S2a–S2e provide a graphical representation of the minimum sample size for three possible impacts, and considers a range of values of intraclass correlations for all outcomes. For example, taking S2a Fig, in blue we see that the minimum sample size needed to detect a difference of one hour of formal services per week. This effect size cannot be substantiated as significant when rho is larger than 0.1 as can be seen in the figure. For smaller rhos there may still need over 100 people per cluster to detect a statistically significant one-hour difference. Given that we have on average five observations per cluster, the SDAC survey would need to be much larger to detect a difference of one hour per week. S2c Fig shows that to detect a 10-percentage-point increase in employment we would only need a sample size of approximately 50 observations in each group even for large intraclass correlations. Generally speaking, the SDAC survey which is currently the only survey capable of evaluating the impact of the NDIS on carers would have benefited from having a substantial increase in sample size prior to the NDIS roll out sot that this multi-billion-dollar scheme could have been properly evaluated.

## Discussion

While one of the goals of the NDIS is to increase personalised formal service use and the wellbeing of the carers, the actual effects on the population level of those with profound or severe disability under the age of 65 are less certain given the replacement of one system with another. Addressing this evidence gap, this study used the staggered roll-out of the NDIS to examine changes in primary carers’ outcomes, including their weekly caring hours and employment and social participation, and investigated if the carer outcome changed due to the change in the use of formal services. It’s important to note that our discussions below were constrained by the limited evidence available due to the small sample size of the SDAC.

We find that formal service use for those people with a severe or profound disability (or needing aids) is similar before and after the NDIS becomes available. As discussed in the introduction, state or territory disability support programs were scaled back when the NDIS became available in the area. This has created challenges for the people who still need to be added to the NDIS but are living in NDIS-available areas and who may have been able to previously access state-based services [5]. In particular, at the early stages of the NDIS implementation, the process of getting an approved plan for the NDIS was so long that even eligible people who had applied would wait for more than seven months [7]. In addition, early research also reported that the supply of disability support services had not developed adequately to satisfy the extra needs created by the NDIS [9], especially in remote areas where the market for formal caring services is thin. Research also shows that NDIS participants with lower socioeconomic status or psychosocial disability, who are Aboriginal and Torres Islanders or who belong to a Cultural and Linguistic Diverse group have lower utilisation rates of the NDIS plan than their counterparts [15,26,27]. This extra barrier of finding and accessing formal care services may result in people with NDIS funding remaining unable to access the services they need, and enhancing cultural awareness among service providers might increase access.

We found no evidence that NDIS availability in an area had short-term impacts on the weekly average caring hours for primary carers of those with a profound or severe disability under 65. The lack of change in informal caring hours may imply that the NDIS has not freed up the carer’s time in the short-term of those who managed to get access to the NDIS; for example, carers may need to spend significant time helping recipient access services for which they have funding. The result could also suggest that any improvements are counteracted by negative impacts for those whose recipients did not get access to the NDIS.

Our study also found no impact of NDIS availability on the employment of primary carers. This is consistent with an early evaluation of the NDIS, which found that the percentage of carers who reported paid work did not change for NDIS participants [10]. The report also showed that the percentage of carers who would like to work more has not changed after their recipients enrolled in NDIS, with the lack of flexibility at work being the key barrier. Our research only examined the effect within two years of NDIS being available, and many primary carers may have already been disconnected from the labour market for many years, making re-engagement difficult. The impact on employment outcomes of primary carers for the NDIS may take many years to be fully realised.

The impact on social participation was expected more immediately than employment. We found no association between NDIS and social participation with or without the care recipient. The lack of change may reflect additional barriers for carers to participate in social events. For example, if the carers do not care for their recipient full time, they may lose their carer payment or receive a reduced amount of carer allowance. The financial pressure may prevent them from joining an event. Additionally, our study only looked at social participation, such as going to a zoo or library or attending a concert or a sports event. Therefore, other common social activities, such as catching up with friends for a coffee or dinner, are not included.

Even though our study focuses on the short-term impact of the NDIS on those potentially eligible NDIS participants, it provides a reference for a broader policy issue. As of March 2021, about 450,000 Australians are on the NDIS, representing just 10% of the people with disability in Australia. These “ineligible” people with disability are not able to access the scheme for various reasons. For instance, people with mental health problems may find it difficult to get into the NDIS because their symptoms are episodic and may or may not be long-term, and it is unclear how their needs during episodes should be met [28]. The evidence required to obtain access to the NDIS is also unclear, and people from a culturally or linguistically diverse background may have less understanding of the system and be less able to advocate for themselves. While aiming to provide appropriate services to the people on the NDIS, it is also critical to consider how to best assist those on the margin of NDIS access and their carers.

### Limitation

Our research results are robust to different sample restrictions. However, there are several limitations. Even with some of the best available data on those with a disability, we suffer from the lack of statistical power in our analysis, which makes it susceptible to Type II error, or we are less likely to detect small changes in the outcomes accurately. Our sample size power calculation indicates that a much larger sample size would have been required to evaluate the NDIS using the same strategy. Future evaluations might usefully consider linked administrative data to be able to detect meaningful differences due to the NDIS implementations. However, such analyse will not be able to cover as broad a range of outcomes as the SDAC and ideally SDAC would have been increased prior to the NDIS implementation. Second, due to data limitations, we have only two time points and, therefore, cannot test the ‘parallel trend’ assumption in the pre-treatment period. However, our results are still robust when using alternative control groups. Third, we used a repeated cross-sectional dataset in our analysis, recruiting new respondents in each wave of the survey, which could impact the results if the composition of the samples changed. Not following up with the same population over time also means we cannot apply individual fixed effects in the model to control for time-invariant individual characteristics. However, we compared the characteristics of primary carers and their recipients of care in these two waves and controlled for the potential differences in these characteristics in our analysis. We have also conducted robustness checks with a matched sample and found similar results. We also explored the potential heterogeneity of the impact of NDIS, but we did not find any effect. Lastly, we do not have the NDIS utilisation data available, so we cannot account for the different impacts the utilisation rate may bring. But since the sample comprises people with disability on and not on the NDIS, we have applied the LGA fixed effects to control for areas characteristics that are stable over time, including factors on the supply side.

## Conclusion

While these limitations should be addressed in future studies, our current analysis of the roll-out of the NDIS between 2015 and 2018 suggests that we lack sufficient evidence to confirm that the new support system is making positive changes for primary carers of individuals under age 65 with profound or severe disability. The use of formal care services at a population level has not increased in the short term. While previous evaluations found positive impacts of the NDIS on the enrolled individual [11], we do not see positive spill-over effects on the carer’s caring time, social participation and employment. Our findings suggest that there may be a need not only to continue improving the support for people with disability on the NDIS but also to provide support for those on the margin of the NDIS. Specific consideration should also be given to the carers, for example, to provide them with more work flexibility so they can balance work and caring responsibilities.

### Further research

Future research should be conducted to evaluate the long-term effect of the NDIS when data becomes available. It is worth exploring further how we should support people with disability not on the NDIS in parallel with the NDIS. Access to disability services, thin markets and cultural awareness among service providers in Australia and also in international context are some topics that are worth exploring in future. Longitudinal data, and administrative data connecting with survey data capturing a wider range of outcomes should also be employed. The recent development of the National Disability Data Asset (NDDA) in Australia provides a unique opportunity to have a more complete picture of the programs and services people with disability use and thereby help better understand the overall impact of the NDIS as well as other policies aim at better supporting people with disability and their carers. Future research could also incorporate qualitative methods, such as interviews and focus groups, to gain deeper insights into the personal experiences and challenges faced by individuals accessing disability services, thereby complementing and enriching the quantitative findings and offering more insights into potential policy implications.

## Supporting information

S1 Table The NDIS trials and rollout. (DOCX)

S2 Table Eligibility Criteria for the trial, treatment period, and control period. (DOCX)

S3 Table Description of the constructed variables. (DOCX)

S4 Table Comparison of outcome and characteristics among the participants and non-participants observed in 2015 and 2018. (DOCX)

S5 Table Main Analysis - complete table. (DOCX)

S6 Table The impact of NDIS on social participation of recipients. (DOCX)

S7 Table Subgroup Analysis (15–24 vs other ages). (DOCX)

S8 Table Sensitivity analysis: weighted sample. (DOCX)

S9 Table Sensitivity analysis: Regional fixed effects. (DOCX)

S10 Table Sensitivity analysis: Cut-off date at 30 June 2017. (DOCX)

S11 Table Sensitivity analysis: Main recipients only. (DOCX)

S12 Table Sensitivity Analysis: Excluding carers eligible for NDIS. (DOCX)

S13 Table Sensitivity Analysis—Different control group (not eligible and live in NDIS available areas). (DOCX)

S14 Table Sensitivity Analysis—Margins of Treatment * Post (accounting for characteristics interaction with the treatment* post variable). (DOCX)

S15 Table Placebo analysis on primary carers of non-NDIS-eligible caring recipients. (DOCX)

S1 Fig The flowchart of sample restriction and sample size. (DOCX)

S2a-S2e Fig Results of sample size estimation by outcomes of interest. (DOCX)
