# Supplementary material for: Exploring the impact of a personalised disability reform on people with disability and their primary carers: Evidence from the Australian national disability insurance scheme
Source: PLoS One. 2025 May 7;20(5):e0321377. doi: 10.1371/journal.pone.0321377 (PMC12057950; doi:10.1371/journal.pone.0321377)
Supplement: S1 Table — (DOCX) [file pone.0321377.s001.docx]

### Table S1: The NDIS trials and rollout

The NDIS is funded by both the Commonwealth and the state and territory governments. There are several stages of the implementation of the NDIS. It was introduced on 1 July 2013 by the Gillard government and first tested in trial sites from 2013. The trial sites include:

- Tasmania for young people aged 15-24;
- South Australia for children aged under 14;
- the Barwon area of Victoria;
- the Hunter area in New South Wales for people up to age 65;
- ACT (started in 2014);
- Barkley region in the Northern Territory (started in 2014).

The following table lists the NDIS starting date in each LGAs and Regions in Mainland Australia (WA excluded).

| **LGA_2014** | **AREA/Region** | **State** | **NDIS Start** |
| --- | --- | --- | --- |
| Gosford (C) | Central Coast | NSW | 1/07/2016 |
| Wyong (A) | Central Coast | NSW | 1/07/2016 |
| Balranald (A) | Far West | NSW | 1/07/2017 |
| Broken Hill (C) | Far West | NSW | 1/07/2017 |
| Central Darling (A) | Far West | NSW | 1/07/2017 |
| Wentworth (A) | Far West | NSW | 1/07/2017 |
| Armidale Dumaresq (A) | Hunter New England | NSW | 1/07/2016 |
| Cessnock (C) | Hunter New England | NSW | 1/07/2016 |
| Dungog (A) | Hunter New England | NSW | 1/07/2016 |
| Glen Innes Severn (A) | Hunter New England | NSW | 1/07/2016 |
| Gloucester (A) | Hunter New England | NSW | 1/07/2016 |
| Great Lakes (A) | Hunter New England | NSW | 1/07/2016 |
| Greater Taree (C) | Hunter New England | NSW | 1/07/2016 |
| Gunnedah (A) | Hunter New England | NSW | 1/07/2016 |
| Guyra (A) | Hunter New England | NSW | 1/07/2016 |
| Gwydir (A) | Hunter New England | NSW | 1/07/2016 |
| Inverell (A) | Hunter New England | NSW | 1/07/2016 |
| Lake Macquarie (C) | Hunter New England | NSW | 1/07/2014 |
| Liverpool Plains (A) | Hunter New England | NSW | 1/07/2016 |
| Maitland (C) | Hunter New England | NSW | 1/07/2015 |
| Moree Plains (A) | Hunter New England | NSW | 1/07/2016 |
| Muswellbrook (A) | Hunter New England | NSW | 1/07/2016 |
| Narrabri (A) | Hunter New England | NSW | 1/07/2016 |
| Newcastle (C) | Hunter New England | NSW | 1/07/2013 |
| Port Stephens (A) | Hunter New England | NSW | 1/07/2016 |
| Singleton (A) | Hunter New England | NSW | 1/07/2016 |
| Tamworth Regional (A) | Hunter New England | NSW | 1/07/2016 |
| Tenterfield (A) | Hunter New England | NSW | 1/07/2016 |
| Upper Hunter Shire (A) | Hunter New England | NSW | 1/07/2016 |
| Uralla (A) | Hunter New England | NSW | 1/07/2016 |
| Walcha (A) | Hunter New England | NSW | 1/07/2016 |
| Kiama (A) | Illawarra Shoalhaven | NSW | 1/07/2017 |
| Shellharbour (C) | Illawarra Shoalhaven | NSW | 1/07/2017 |
| Shoalhaven (C) | Illawarra Shoalhaven | NSW | 1/07/2017 |
| Wollongong (C) | Illawarra Shoalhaven | NSW | 1/07/2017 |
| Bellingen (A) | Mid North Coast | NSW | 1/07/2017 |
| Coffs Harbour (C) | Mid North Coast | NSW | 1/07/2017 |
| Kempsey (A) | Mid North Coast | NSW | 1/07/2017 |
| Nambucca (A) | Mid North Coast | NSW | 1/07/2017 |
| Port Macquarie-Hastings (A) | Mid North Coast | NSW | 1/07/2017 |
| Albury (C) | Murrumbidgee | NSW | 1/07/2017 |
| Berrigan (A) | Murrumbidgee | NSW | 1/07/2017 |
| Bland (A) | Murrumbidgee | NSW | 1/07/2017 |
| Boorowa (A) | Murrumbidgee | NSW | 1/07/2017 |
| Carrathool (A) | Murrumbidgee | NSW | 1/07/2017 |
| Conargo (A) | Murrumbidgee | NSW | 1/07/2017 |
| Coolamon (A) | Murrumbidgee | NSW | 1/07/2017 |
| Cootamundra (A) | Murrumbidgee | NSW | 1/07/2017 |
| Corowa Shire (A) | Murrumbidgee | NSW | 1/07/2017 |
| Deniliquin (A) | Murrumbidgee | NSW | 1/07/2017 |
| Greater Hume Shire (A) | Murrumbidgee | NSW | 1/07/2017 |
| Griffith (C) | Murrumbidgee | NSW | 1/07/2017 |
| Gundagai (A) | Murrumbidgee | NSW | 1/07/2017 |
| Harden (A) | Murrumbidgee | NSW | 1/07/2017 |
| Hay (A) | Murrumbidgee | NSW | 1/07/2017 |
| Jerilderie (A) | Murrumbidgee | NSW | 1/07/2017 |
| Junee (A) | Murrumbidgee | NSW | 1/07/2017 |
| Leeton (A) | Murrumbidgee | NSW | 1/07/2017 |
| Lockhart (A) | Murrumbidgee | NSW | 1/07/2017 |
| Murray (A) | Murrumbidgee | NSW | 1/07/2017 |
| Murrumbidgee (A) | Murrumbidgee | NSW | 1/07/2017 |
| Narrandera (A) | Murrumbidgee | NSW | 1/07/2017 |
| Temora (A) | Murrumbidgee | NSW | 1/07/2017 |
| Tumbarumba (A) | Murrumbidgee | NSW | 1/07/2017 |
| Tumut Shire (A) | Murrumbidgee | NSW | 1/07/2017 |
| Urana (A) | Murrumbidgee | NSW | 1/07/2017 |
| Wagga Wagga (C) | Murrumbidgee | NSW | 1/07/2017 |
| Wakool (A) | Murrumbidgee | NSW | 1/07/2017 |
| Young (A) | Murrumbidgee | NSW | 1/07/2017 |
| Blue Mountains (C) | Nepean Blue Mountains | NSW | 1/07/2016 |
| Hawkesbury (C) | Nepean Blue Mountains | NSW | 1/07/2017 |
| Lithgow (C) | Nepean Blue Mountains | NSW | 1/07/2017 |
| Penrith (C) | Nepean Blue Mountains | NSW | 1/07/2017 |
| Ballina (A) | Northern NSW | NSW | 1/07/2017 |
| Byron (A) | Northern NSW | NSW | 1/07/2017 |
| Clarence Valley (A) | Northern NSW | NSW | 1/07/2017 |
| Kyogle (A) | Northern NSW | NSW | 1/07/2017 |
| Lismore (C) | Northern NSW | NSW | 1/07/2017 |
| Richmond Valley (A) | Northern NSW | NSW | 1/07/2017 |
| Tweed (A) | Northern NSW | NSW | 1/07/2017 |
| Hornsby (A) | Northern Sydney | NSW | 1/07/2016 |
| Hunters Hill (A) | Northern Sydney | NSW | 1/07/2016 |
| Ku-ring-gai (A) | Northern Sydney | NSW | 1/07/2016 |
| Lane Cove (A) | Northern Sydney | NSW | 1/07/2016 |
| Manly (A) | Northern Sydney | NSW | 1/07/2016 |
| Mosman (A) | Northern Sydney | NSW | 1/07/2016 |
| North Sydney (A) | Northern Sydney | NSW | 1/07/2016 |
| Pittwater (A) | Northern Sydney | NSW | 1/07/2016 |
| Ryde (C) | Northern Sydney | NSW | 1/07/2016 |
| Warringah (A) | Northern Sydney | NSW | 1/07/2016 |
| Willoughby (C) | Northern Sydney | NSW | 1/07/2016 |
| Botany Bay (C) | South Eastern Sydney | NSW | 1/07/2017 |
| Hurstville (C) | South Eastern Sydney | NSW | 1/07/2017 |
| Kogarah (C) | South Eastern Sydney | NSW | 1/07/2017 |
| Randwick (C) | South Eastern Sydney | NSW | 1/07/2017 |
| Rockdale (C) | South Eastern Sydney | NSW | 1/07/2017 |
| Sutherland Shire (A) | South Eastern Sydney | NSW | 1/07/2017 |
| Sydney (C) | South Eastern Sydney | NSW | 1/07/2017 |
| Waverley (A) | South Eastern Sydney | NSW | 1/07/2017 |
| Woollahra (A) | South Eastern Sydney | NSW | 1/07/2017 |
| Bankstown (C) | South Western Sydney | NSW | 1/07/2016 |
| Camden (A) | South Western Sydney | NSW | 1/07/2016 |
| Campbelltown (C) | South Western Sydney | NSW | 1/07/2016 |
| Fairfield (C) | South Western Sydney | NSW | 1/07/2016 |
| Liverpool (C) | South Western Sydney | NSW | 1/07/2016 |
| Wingecarribee (A) | South Western Sydney | NSW | 1/07/2016 |
| Wollondilly (A) | South Western Sydney | NSW | 1/07/2016 |
| Bega Valley (A) | Southern NSW | NSW | 1/07/2016 |
| Bombala (A) | Southern NSW | NSW | 1/07/2016 |
| Cooma-Monaro (A) | Southern NSW | NSW | 1/07/2016 |
| Eurobodalla (A) | Southern NSW | NSW | 1/07/2016 |
| Goulburn Mulwaree (A) | Southern NSW | NSW | 1/07/2016 |
| Palerang (A) | Southern NSW | NSW | 1/07/2016 |
| Queanbeyan (C) | Southern NSW | NSW | 1/07/2016 |
| Snowy River (A) | Southern NSW | NSW | 1/07/2016 |
| Upper Lachlan Shire (A) | Southern NSW | NSW | 1/07/2016 |
| Yass Valley (A) | Southern NSW | NSW | 1/07/2016 |
| Ashfield (A) | Sydney | NSW | 1/07/2017 |
| Burwood (A) | Sydney | NSW | 1/07/2017 |
| Canada Bay (A) | Sydney | NSW | 1/07/2017 |
| Canterbury (C) | Sydney | NSW | 1/07/2017 |
| Leichhardt (A) | Sydney | NSW | 1/07/2017 |
| Marrickville (A) | Sydney | NSW | 1/07/2017 |
| Strathfield (A) | Sydney | NSW | 1/07/2017 |
| Sydney (C) | Sydney | NSW | 1/07/2017 |
| Bathurst Regional (A) | Western NSW | NSW | 1/07/2017 |
| Blayney (A) | Western NSW | NSW | 1/07/2017 |
| Bogan (A) | Western NSW | NSW | 1/07/2017 |
| Bourke (A) | Western NSW | NSW | 1/07/2017 |
| Brewarrina (A) | Western NSW | NSW | 1/07/2017 |
| Cabonne (A) | Western NSW | NSW | 1/07/2017 |
| Cobar (A) | Western NSW | NSW | 1/07/2017 |
| Coonamble (A) | Western NSW | NSW | 1/07/2017 |
| Cowra (A) | Western NSW | NSW | 1/07/2017 |
| Dubbo (C) | Western NSW | NSW | 1/07/2017 |
| Forbes (A) | Western NSW | NSW | 1/07/2017 |
| Gilgandra (A) | Western NSW | NSW | 1/07/2017 |
| Lachlan (A) | Western NSW | NSW | 1/07/2017 |
| Mid-Western Regional (A) | Western NSW | NSW | 1/07/2017 |
| Narromine (A) | Western NSW | NSW | 1/07/2017 |
| Oberon (A) | Western NSW | NSW | 1/07/2017 |
| Orange (C) | Western NSW | NSW | 1/07/2017 |
| Parkes (A) | Western NSW | NSW | 1/07/2017 |
| Walgett (A) | Western NSW | NSW | 1/07/2017 |
| Warren (A) | Western NSW | NSW | 1/07/2017 |
| Warrumbungle Shire (A) | Western NSW | NSW | 1/07/2017 |
| Weddin (A) | Western NSW | NSW | 1/07/2017 |
| Wellington (A) | Western NSW | NSW | 1/07/2017 |
| Auburn (C) | Western Sydney | NSW | 1/07/2017 |
| Blacktown (C) | Western Sydney | NSW | 1/07/2017 |
| Holroyd (C) | Western Sydney | NSW | 1/07/2017 |
| Parramatta (C) | Western Sydney | NSW | 1/07/2017 |
| The Hills Shire (A) | Western Sydney | NSW | 1/07/2017 |
| Bayside (C) | Bayside Peninsula | VIC | 1/04/2018 |
| Frankston (C) | Bayside Peninsula | VIC | 1/04/2018 |
| Glen Eira (C) | Bayside Peninsula | VIC | 1/04/2018 |
| Kingston (C) | Bayside Peninsula | VIC | 1/04/2018 |
| Mornington Peninsula (S) | Bayside Peninsula | VIC | 1/04/2018 |
| Port Phillip (C) | Bayside Peninsula | VIC | 1/04/2018 |
| Stonnington (C) | Bayside Peninsula | VIC | 1/04/2018 |
| Brimbank (C) | Brimbank Melton | VIC | 1/10/2018 |
| Melton (S) | Brimbank Melton | VIC | 1/10/2018 |
| Ararat (RC) | Central Highlands | VIC | 1/01/2017 |
| Ballarat (C) | Central Highlands | VIC | 1/01/2017 |
| Golden Plains (S) | Central Highlands | VIC | 1/01/2017 |
| Hepburn (S) | Central Highlands | VIC | 1/01/2017 |
| Moorabool (S) | Central Highlands | VIC | 1/01/2017 |
| Pyrenees (S) | Central Highlands | VIC | 1/01/2017 |
| Greater Shepparton (C) | Goulburn | VIC | 1/01/2019 |
| Mitchell (S) | Goulburn | VIC | 1/01/2019 |
| Moira (S) | Goulburn | VIC | 1/01/2019 |
| Murrindindi (S) | Goulburn | VIC | 1/01/2019 |
| Strathbogie (S) | Goulburn | VIC | 1/01/2019 |
| Hume (C) | Hume Moreland | VIC | 1/03/2018 |
| Moreland (C) | Hume Moreland | VIC | 1/03/2018 |
| Boroondara (C) | Inner Eastern Melbourne | VIC | 1/11/2017 |
| Manningham (C) | Inner Eastern Melbourne | VIC | 1/11/2017 |
| Monash (C) | Inner Eastern Melbourne | VIC | 1/11/2017 |
| Whitehorse (C) | Inner Eastern Melbourne | VIC | 1/11/2017 |
| Bass Coast (S) | Inner Gippsland | VIC | 1/10/2017 |
| Baw Baw (S) | Inner Gippsland | VIC | 1/10/2017 |
| Latrobe (C) | Inner Gippsland | VIC | 1/10/2017 |
| South Gippsland (S) | Inner Gippsland | VIC | 1/10/2017 |
| Campaspe (S) | Loddon | VIC | 1/05/2017 |
| Central Goldfields (S) | Loddon | VIC | 1/05/2017 |
| Greater Bendigo(C ) | Loddon | VIC | 1/05/2017 |
| Loddon (S) | Loddon | VIC | 1/05/2017 |
| Macedon Ranges (S) | Loddon | VIC | 1/05/2017 |
| Mount Alexander (S) | Loddon | VIC | 1/05/2017 |
| Buloke (S) | Mallee | VIC | 1/01/2019 |
| Gannawarra (S) | Mallee | VIC | 1/01/2019 |
| Mildura (RC) | Mallee | VIC | 1/01/2019 |
| Swan Hill (RC) | Mallee | VIC | 1/01/2019 |
| Banyule (C) | North Eastern Melbourne | VIC | 1/07/2016 |
| Darebin (C) | North Eastern Melbourne | VIC | 1/07/2016 |
| Nillumbik (S) | North Eastern Melbourne | VIC | 1/07/2016 |
| Whittlesea (C) | North Eastern Melbourne | VIC | 1/07/2016 |
| Yarra (C) | North Eastern Melbourne | VIC | 1/07/2016 |
| Knox (C) | Outer Eastern Melbourne | VIC | 1/11/2017 |
| Maroondah (C) | Outer Eastern Melbourne | VIC | 1/11/2017 |
| Yarra Ranges (S) | Outer Eastern Melbourne | VIC | 1/11/2017 |
| East Gippsland (S) | Outer Gippsland | VIC | 1/01/2019 |
| Wellington (S) | Outer Gippsland | VIC | 1/01/2019 |
| Alpine (S) | Ovens Murray | VIC | 1/10/2017 |
| Benalla (RC) | Ovens Murray | VIC | 1/10/2017 |
| Indigo (S) | Ovens Murray | VIC | 1/10/2017 |
| Mansfield (S) | Ovens Murray | VIC | 1/10/2017 |
| Towong (S) | Ovens Murray | VIC | 1/10/2017 |
| Wangaratta (RC) | Ovens Murray | VIC | 1/10/2017 |
| Wodonga (RC) | Ovens Murray | VIC | 1/10/2017 |
| Cardinia (S) | South Melbourne | VIC | 1/09/2018 |
| Casey (C) | South Melbourne | VIC | 1/09/2018 |
| Greater Dandenong (C) | South Melbourne | VIC | 1/09/2018 |
| Corangamite (S) | Western District | VIC | 1/10/2017 |
| Glenelg (S) | Western District | VIC | 1/10/2017 |
| Hindmarsh (S) | Western District | VIC | 1/10/2017 |
| Horsham (RC) | Western District | VIC | 1/10/2017 |
| Moyne (S) | Western District | VIC | 1/10/2017 |
| Northern Grampians (S) | Western District | VIC | 1/10/2017 |
| Southern Grampians (S) | Western District | VIC | 1/10/2017 |
| Warrnambool (C) | Western District | VIC | 1/10/2017 |
| West Wimmera (S) | Western District | VIC | 1/10/2017 |
| Yarriambiack (S) | Western District | VIC | 1/10/2017 |
| Hobsons Bay (C) | Western Melbourne | VIC | 1/10/2018 |
| Maribyrnong (C) | Western Melbourne | VIC | 1/10/2018 |
| Melbourne (C) | Western Melbourne | VIC | 1/10/2018 |
| Moonee Valley (C) | Western Melbourne | VIC | 1/10/2018 |
| Wyndham (C) | Western Melbourne | VIC | 1/10/2018 |
| Colac-Otway (S) | Barwon | VIC | 1/07/2013 |
| Greater Geelong (C) | Barwon | VIC | 1/07/2013 |
| Surf Coast (S) | Barwon | VIC | 1/07/2013 |
| Queenscliffe (B) | Barwon | VIC | 1/07/2018 |
| Logan (C) | Beenleigh | QLD | 1/07/2018 |
| Redland (C) | Beenleigh | QLD | 1/07/2018 |
| Brisbane(C ) | Brisbane North | QLD | 1/07/2018 |
| Bundaberg (R) | Bundaberg | QLD | 1/10/2017 |
| Moreton Bay (R) | Caboolture/Strathpine | QLD | 1/01/2019 |
| Aurukun (S) | Cairns | QLD | 1/07/2018 |
| Cairns (R) | Cairns | QLD | 1/07/2018 |
| Cassowary Coast (R) | Cairns | QLD | 1/07/2018 |
| Cook (S) | Cairns | QLD | 1/07/2018 |
| Croydon (S) | Cairns | QLD | 1/07/2018 |
| Douglas (S) | Cairns | QLD | 1/07/2018 |
| Etheridge (S) | Cairns | QLD | 1/07/2018 |
| Hope Vale (S) | Cairns | QLD | 1/07/2018 |
| Kowanyama (S) | Cairns | QLD | 1/07/2018 |
| Lockhart River (S) | Cairns | QLD | 1/07/2018 |
| Mapoon (S) | Cairns | QLD | 1/07/2018 |
| Mareeba (S) | Cairns | QLD | 1/07/2018 |
| Napranum (S) | Cairns | QLD | 1/07/2018 |
| Northern Peninsula Area (R) | Cairns | QLD | 1/07/2018 |
| Pormpuraaw (S) | Cairns | QLD | 1/07/2018 |
| Tablelands (R) | Cairns | QLD | 1/07/2018 |
| Torres (S) | Cairns | QLD | 1/07/2018 |
| Torres Strait Island (R) | Cairns | QLD | 1/07/2018 |
| Weipa (T) | Cairns | QLD | 1/07/2018 |
| Wujal Wujal (S) | Cairns | QLD | 1/07/2018 |
| Yarrabah (S) | Cairns | QLD | 1/07/2018 |
| Ipswich (C) | Ipswich | QLD | 1/07/2017 |
| Lockyer Valley (R) | Ipswich | QLD | 1/07/2017 |
| Scenic Rim (R) | Ipswich | QLD | 1/07/2017 |
| Somerset (R) | Ipswich | QLD | 1/07/2017 |
| Isaac (R) | Mackay | QLD | 1/11/2016 |
| Mackay (R) | Mackay | QLD | 1/11/2016 |
| Whitsunday (R) | Mackay | QLD | 1/11/2016 |
| Gympie (R) | Maroochydore | QLD | 1/01/2019 |
| Noosa (S) | Maroochydore | QLD | 1/01/2019 |
| Sunshine Coast (R) | Maroochydore | QLD | 1/01/2019 |
| Cherbourg (S) | Maryborough | QLD | 1/07/2018 |
| Fraser Coast (R) | Maryborough | QLD | 1/07/2018 |
| North Burnett (R) | Maryborough | QLD | 1/07/2018 |
| South Burnett (R) | Maryborough | QLD | 1/07/2018 |
| Gold Coast (C) | Robina | QLD | 1/07/2018 |
| Banana (S) | Rockhampton | QLD | 1/01/2018 |
| Barcaldine (R) | Rockhampton | QLD | 1/01/2018 |
| Barcoo (S) | Rockhampton | QLD | 1/01/2018 |
| Blackall Tambo (R) | Rockhampton | QLD | 1/01/2018 |
| Central Highlands (R) | Rockhampton | QLD | 1/01/2018 |
| Diamantina (S) | Rockhampton | QLD | 1/01/2018 |
| Gladstone (R) | Rockhampton | QLD | 1/01/2018 |
| Livingstone (S) | Rockhampton | QLD | 1/01/2018 |
| Longreach (R) | Rockhampton | QLD | 1/01/2018 |
| Rockhampton (R) | Rockhampton | QLD | 1/01/2018 |
| Winton (S) | Rockhampton | QLD | 1/01/2018 |
| Woorabinda (S) | Rockhampton | QLD | 1/01/2018 |
| Balonne (S) | Toowoomba | QLD | 1/01/2017 |
| Bulloo (S) | Toowoomba | QLD | 1/01/2017 |
| Goondiwindi (R) | Toowoomba | QLD | 1/01/2017 |
| Maranoa (R) | Toowoomba | QLD | 1/01/2017 |
| Murweh (S) | Toowoomba | QLD | 1/01/2017 |
| Paroo (S) | Toowoomba | QLD | 1/01/2017 |
| Quilpie (S) | Toowoomba | QLD | 1/01/2017 |
| Southern Downs (R) | Toowoomba | QLD | 1/01/2017 |
| Toowoomba (R) | Toowoomba | QLD | 1/01/2017 |
| Western Downs (R) | Toowoomba | QLD | 1/01/2017 |
| Boulia (S) | Townsville | QLD | 1/10/2016 |
| Burdekin (S) | Townsville | QLD | 1/10/2016 |
| Burke (S) | Townsville | QLD | 1/10/2016 |
| Carpentaria (S) | Townsville | QLD | 1/10/2016 |
| Charters Towers (R) | Townsville | QLD | 1/01/2016 |
| Cloncurry (S) | Townsville | QLD | 1/10/2016 |
| Doomadgee (S) | Townsville | QLD | 1/10/2016 |
| Flinders (S) | Townsville | QLD | 1/10/2016 |
| Hinchinbrook (S) | Townsville | QLD | 1/10/2016 |
| McKinlay (S) | Townsville | QLD | 1/10/2016 |
| Mornington (S) | Townsville | QLD | 1/10/2016 |
| Mount Isa (C) | Townsville | QLD | 1/10/2016 |
| Palm Island (S) | Townsville | QLD | 1/10/2016 |
| Richmond (S) | Townsville | QLD | 1/10/2016 |
| Townsville (C) | Townsville | QLD | 1/01/2016 |
| Adelaide Hills (DC) | Adelaide Hills | SA | 1/04/2018 |
| Mount Barker (DC) | Adelaide Hills | SA | 1/04/2018 |
| Barossa (DC) | Barossa, Light and Lower North | SA | 1/07/2017 |
| Gawler (T) | Barossa, Light and Lower North | SA | 1/07/2017 |
| Light (RegC) | Barossa, Light and Lower North | SA | 1/07/2017 |
| Mallala (DC) | Barossa, Light and Lower North | SA | 1/07/2017 |
| Adelaide (C) | Eastern Adelaide | SA | 1/04/2018 |
| Burnside (C) | Eastern Adelaide | SA | 1/04/2018 |
| Campbelltown (C) | Eastern Adelaide | SA | 1/04/2018 |
| Norwood Payneham St Peters (C) | Eastern Adelaide | SA | 1/04/2018 |
| Prospect (C) | Eastern Adelaide | SA | 1/04/2018 |
| Unley (C) | Eastern Adelaide | SA | 1/04/2018 |
| Walkerville (M) | Eastern Adelaide | SA | 1/04/2018 |
| Ceduna (DC) | Eyre and Western | SA | 1/01/2018 |
| Cleve (DC) | Eyre and Western | SA | 1/01/2018 |
| Elliston (DC) | Eyre and Western | SA | 1/01/2018 |
| Franklin Harbour (DC) | Eyre and Western | SA | 1/01/2018 |
| Kimba (DC) | Eyre and Western | SA | 1/01/2018 |
| Lower Eyre Peninsula (DC) | Eyre and Western | SA | 1/01/2018 |
| Port Lincoln (C) | Eyre and Western | SA | 1/01/2018 |
| Streaky Bay (DC) | Eyre and Western | SA | 1/01/2018 |
| Tumby Bay (DC) | Eyre and Western | SA | 1/01/2018 |
| Whyalla (C) | Eyre and Western | SA | 1/01/2018 |
| Wudinna (DC) | Eyre and Western | SA | 1/01/2018 |
| Anangu Pitjantjatjara (AC) | Far North | SA | 1/01/2018 |
| Coober Pedy (DC) | Far North | SA | 1/01/2018 |
| Flinders Ranges (DC) | Far North | SA | 1/01/2018 |
| Port Augusta (C) | Far North | SA | 1/01/2018 |
| Roxby Downs (M) | Far North | SA | 1/01/2018 |
| Alexandrina (DC) | Fleurier and Kangaroo Island | SA | 1/01/2018 |
| Kangaroo Island (DC) | Fleurier and Kangaroo Island | SA | 1/01/2018 |
| Victor Harbor (C) | Fleurier and Kangaroo Island | SA | 1/01/2018 |
| Yankalilla (DC) | Fleurier and Kangaroo Island | SA | 1/01/2018 |
| Grant (DC) | Limestone Coast | SA | 1/10/2017 |
| Kingston (DC) | Limestone Coast | SA | 1/10/2017 |
| Mount Gambier (C) | Limestone Coast | SA | 1/10/2017 |
| Naracoorte and Lucindale (DC) | Limestone Coast | SA | 1/10/2017 |
| Robe (DC) | Limestone Coast | SA | 1/10/2017 |
| Tatiara (DC) | Limestone Coast | SA | 1/10/2017 |
| Wattle Range (DC) | Limestone Coast | SA | 1/10/2017 |
| Berri and Barmera (DC) | Murray and Mallee | SA | 1/10/2017 |
| Karoonda East Murray (DC) | Murray and Mallee | SA | 1/10/2017 |
| Loxton Waikerie (DC) | Murray and Mallee | SA | 1/10/2017 |
| Mid Murray (DC) | Murray and Mallee | SA | 1/10/2017 |
| Murray Bridge (RC) | Murray and Mallee | SA | 1/10/2017 |
| Renmark Paringa (DC) | Murray and Mallee | SA | 1/10/2017 |
| Southern Mallee (DC) | Murray and Mallee | SA | 1/10/2017 |
| The Coorong (DC) | Murray and Mallee | SA | 1/10/2017 |
| Playford (C) | Northern Adelaide | SA | 1/07/2017 |
| Port Adelaide Enfield (C) | Northern Adelaide | SA | 1/07/2017 |
| Salisbury (C) | Northern Adelaide | SA | 1/07/2017 |
| Tea Tree Gully (C) | Northern Adelaide | SA | 1/10/2017 |
| Holdfast Bay (C) | Southern Adelaide | SA | 1/01/2018 |
| Marion (C) | Southern Adelaide | SA | 1/01/2018 |
| Mitcham (C) | Southern Adelaide | SA | 1/01/2018 |
| Onkaparinga (C) | Southern Adelaide | SA | 1/01/2018 |
| Charles Sturt (C) | Western Adelaide | SA | 1/04/2018 |
| Port Adelaide Enfield (C) | Western Adelaide | SA | 1/04/2018 |
| West Torrens (C) | Western Adelaide | SA | 1/04/2018 |
| Barunga West (DC) | Yorke and Mid North | SA | 1/01/2018 |
| Clare and Gilbert Valleys (DC) | Yorke and Mid North | SA | 1/01/2018 |
| Copper Coast (DC) | Yorke and Mid North | SA | 1/01/2018 |
| Goyder (DC) | Yorke and Mid North | SA | 1/01/2018 |
| Mount Remarkable (DC) | Yorke and Mid North | SA | 1/01/2018 |
| Northern Areas (DC) | Yorke and Mid North | SA | 1/01/2018 |
| Orroroo/Carrieton (DC) | Yorke and Mid North | SA | 1/01/2018 |
| Peterborough (DC) | Yorke and Mid North | SA | 1/01/2018 |
| Port Pirie City and Dists (M) | Yorke and Mid North | SA | 1/01/2018 |
| Wakefield (DC) | Yorke and Mid North | SA | 1/01/2018 |
| Yorke Peninsula (DC) | Yorke and Mid North | SA | 1/01/2018 |
| Break O'Day (M) | NA | TAS | 1/07/2016 |
| Brighton (M) | NA | TAS | 1/07/2016 |
| Burnie (C) | NA | TAS | 1/07/2016 |
| Central Coast (M) | NA | TAS | 1/07/2016 |
| Central Highlands (M) | NA | TAS | 1/07/2016 |
| Circular Head (M) | NA | TAS | 1/07/2016 |
| Clarence (C) | NA | TAS | 1/07/2016 |
| Derwent Valley (M) | NA | TAS | 1/07/2016 |
| Devonport (C) | NA | TAS | 1/07/2016 |
| Dorset (M) | NA | TAS | 1/07/2016 |
| Flinders (M) | NA | TAS | 1/07/2016 |
| George Town (M) | NA | TAS | 1/07/2016 |
| Glamorgan/Spring Bay (M) | NA | TAS | 1/07/2016 |
| Glenorchy (C) | NA | TAS | 1/07/2016 |
| Hobart (C) | NA | TAS | 1/07/2016 |
| Huon Valley (M) | NA | TAS | 1/07/2016 |
| Kentish (M) | NA | TAS | 1/07/2016 |
| King Island (M) | NA | TAS | 1/07/2016 |
| Kingborough (M) | NA | TAS | 1/07/2016 |
| Latrobe (M) | NA | TAS | 1/07/2016 |
| Launceston (C) | NA | TAS | 1/07/2016 |
| Meander Valley (M) | NA | TAS | 1/07/2016 |
| Northern Midlands (M) | NA | TAS | 1/07/2016 |
| Sorell (M) | NA | TAS | 1/07/2016 |
| Southern Midlands (M) | NA | TAS | 1/07/2016 |
| Tasman (M) | NA | TAS | 1/07/2016 |
| Waratah/Wynyard (M) | NA | TAS | 1/07/2016 |
| West Coast (M) | NA | TAS | 1/07/2016 |
| West Tamar (M) | NA | TAS | 1/07/2016 |
| Barkly (S) | Barkly | NT | 1/07/2014 |
| East Arnhem (S) | East Arnhem | NT | 1/01/2017 |
| Alice Springs (T) | Central Australia | NT | 1/07/2018 |
| Central Desert (S) | Central Australia | NT | 1/07/2018 |
| MacDonnell (S) | Central Australia | NT | 1/07/2018 |
| Belyuen (S) | Darwin Urban | NT | 1/07/2018 |
| Coomalie (S) | Darwin Urban | NT | 1/07/2018 |
| Darwin (C) | Darwin Urban | NT | 1/07/2018 |
| Litchfield (M) | Darwin Urban | NT | 1/07/2018 |
| Palmerston (C) | Darwin Urban | NT | 1/07/2018 |
| Wagait (S) | Darwin Urban | NT | 1/07/2018 |
| Roper Gulf (S) | Darwin Remote | NT | 1/07/2017 |
| Tiwi Islands (S) | Darwin Remote | NT | 1/07/2017 |
| Victoria Daly (R) | Darwin Remote | NT | 1/07/2017 |
| West Arnhem (S) | Darwin Remote | NT | 1/07/2017 |
| West Daly (R) | Darwin Remote | NT | 1/07/2017 |
| Katherine (T) | Katherine | NT | 1/07/2017 |
| Unincorporated ACT | NA | ACT | 1/01/2016 |

Source: The National Disability Insurance Agency: Intergovenmental agreements.
