# Supplementary material for: Exploring the impact of a personalised disability reform on people with disability and their primary carers: Evidence from the Australian national disability insurance scheme
Source: PLoS One. 2025 May 7;20(5):e0321377. doi: 10.1371/journal.pone.0321377 (PMC12057950; doi:10.1371/journal.pone.0321377)
Supplement: S2 Table — (DOCX) [file pone.0321377.s002.docx]

### Table S2: Eligibility Criteria for the trial, treatment period, and control period

| **Trial** | **Trial and rollout method** | **Age** | **Age at data collection** | **Disability** | **Start date** | **Trial  End date** |
| --- | --- | --- | --- | --- | --- | --- |
| **NSW (Hunter) VIC (Barwon)** | geographic (LGA) | <65 | <67 (W15) <70 (W18) | Profound or Severe disability or need aid | 1-Jul-13 | 31-Dec-15 |
| **NSW (Blue Mountain)** | LGA | 0-17 | 0<=age≤18 (W15) 3<=age<=21 (W18) |  | 1-Jul-15 |  |
| **NT  (Barkely)** | LGA | <65 | <66 (W15) <69 (W18) |  | 1-Jul-13 |  |
| **ACT** | Age | 50+3m-65 | 50<=age<=66 (w15) 53<=age<=69 (W18) |  | 1-Jul-14 |  |
|  |  | <=18 | <=19 (W15) <=22 (W18) |  | 1-Apr-15 |  |
| **SA** | AGE | 0-5 | 0<=age<=15 (W15) 3<=age<=18 (W18) |  | 1-Jul-13 |  |
|  |  | 0-13 |  |  | 1-Jul-14 |  |
|  |  | 0-14 |  |  | 1-Jul-15 |  |
| **TAS** | AGE | 15-24 | 15<=age<= 26 (W15) 18<=age<=29 (W18) |  | 1-Jul-13 |  |

| **Treatment** | **Age** | **Age at data collection** | **Disability** | **Starting date within treatment period** | **Treatment period** |
| --- | --- | --- | --- | --- | --- |
| **NSW VIC NT QLD** | <65 | <64 (W15) <67 (W18) | Profound or Severe disability or need aid | 1-Jul-16 | 1-Jan-16 to 30-Jun-18 |
| **SA** | <65 |  |  | 1-Feb-16 |  |
| **ACT** | 28-50 |  |  | 1-Jan-16 |  |
|  | <65 |  |  | 1-Jul-16 |  |
| **TAS** | 12-14 | W15:2<=age<=12 23<=age<=32 W18:  4<=age<=15 25<=age<=35 |  | 1-Jul-16 |  |
|  | 25-28 |  |  | 1-Jan-17 |  |
|  | 4-11 |  |  | 1-Jul-17 |  |
|  | 29-34 |  |  | 1-Jan-18 |  |

| **Control** | **Age** | **Age at data collection** | **Disability** | **Starting date** | **Control period started** |
| --- | --- | --- | --- | --- | --- |
| **VIC ACT  QLD NT** | <65 | <62 (W15) <66 (W18) | Profound or Severe disability or need aid | 1-Jul-18 | 1-Jul-18 |
| **TAS** | 0-3 | W15: 0 32<=age<=61 W18: 0<=age<=4 35<=age<=64 |  | 1-Jul-18 |  |
|  | 35-49 |  |  | 1-Jul-18 |  |
|  | 50-64 |  |  | 1-Jan-19 |  |
