# Supplementary material for: Exploring the impact of a personalised disability reform on people with disability and their primary carers: Evidence from the Australian national disability insurance scheme
Source: PLoS One. 2025 May 7;20(5):e0321377. doi: 10.1371/journal.pone.0321377 (PMC12057950; doi:10.1371/journal.pone.0321377)
Supplement: S3 Table — (DOCX) [file pone.0321377.s003.docx]

### Table S3: Description of the constructed variables

| **Variables** | **Categories of the variable**  **(if applicable)** | **Description** |
| --- | --- | --- |
| **Outcome** | | |
| Weekly caring hours | Continuous variable | Used to describe the number of caring hours per week for primary carers. Converted to categorical variable by taking the midpoint. Original variable is categorised as follows: 9 hours, 10-19 hours, 20-29 hours, 30-39 hours, 40-59 hours, and 60+ hours. |
| Employed FT/PT | 0. Not employed / Not in labour force 1. Employed FT/PT | Used to describe the labour force status of the primary carers. Recoded from the original five-level variable. |
| Social Participation (alone) | 0. Have not participated in social/ cultural events without the recipient of care  / Not leave home in the last 12 months 1. Have participated in social in social/ cultural events without the recipient of care in the last 12 months | Used to describe if the carer had social participation without recipient of care in the last 12 months. Recoded and combined from the 7 original variables.  The types of social events or places include: a public library, a museum or art gallery, a botanic garden, zoo or aquarium, a movie, concert, theatre or other performing arts event, participated in physical activities for sport, participated in physical activities for exercise or recreation, and attended a sporting event as a spectator |
| Social Participation (Any) | 0. Have not participated in social/ cultural events  / Not leave home in the last 12 months 1. Have participated in social in social/ cultural events with/without the recipient of care in the last 12 months | Used to describe if the carer had any social participation in the last 12 months. Recoded and combined from the 7 original variables |
| Extensive margin of formal services use | 0. Have not used formal services in the last 12 months.  1. Have used at least one formal service in the last 12 months | Use to describe if the people with disability have used formal services in the last 12 months. The formal services included are: communication, mobility, selfcare, cognitive tasks, household chores, meal preparation, property maintenance, reading or writing, and transportation |
| Intensive of formal services use | Continuous variable if they have used at least one formal service in the last year. Measured in the number of times of usage/week. | Use to describe the frequency of formal services used by people with disability if they have used any in the last 12 months. |
| Overall use of formal services | Continuous variable including those who have not used any formal services in the last 12 months. Measured in the number of times of usage/week. | Use to describe the overall frequency of formal services used by people with disability regardless of whether they have used any in the last 12 months. |
| **Carer Characteristics** | | |
| Age | Continuous | Age of the primary carers. |
| Male | 0. Female 1. Male | Sex of the primary carers. |
| Number of recipients of carer | Continuous | Number of recipient(s) of care for primary carers Unchanged. |
| Adults without disability in household | Continuous | Number of adults (aged >=15 yo) without disability in the household Constructed by merging the household dataset to the person-level dataset, generated by household ID. |
| Highest education level | 0. Year 11 and below 1. Bachelor and above 2. Certificates/ diploma 3. Year 12 | The highest education level for primary carers. Recoded from the original education variable. The variable was originally asked to people>=15, but we coded people <15 to category 0 as we assume that they are year 11 and below |
| **Recipient of care** | | |
| Age | Continuous | Age of the recipients of care. |
| Male | 0. Female 1. Male | Sex of the recipients of care. |
| Married/ De facto | 0. Single 1. Married/ De facto | Marital status of recipient of care |
| Disability Status | 0. Has disability and severely restricted 1. Has disability and profoundly restricted | Disability status of recipient of care Keep only the severely or proudly restricted respondents. |
| Disability type | 0. Other disabilities  1. Psychosocial disability | Psychosocial disability versus other disability types |
| Born in Australia | 0. Not born in Australia Mainland 1. Born in Australia Mainland | Used to described if the recipient of care was born in Australian Mainland |
| Rurality | 0. Major Cities  1. Inner regional  2. Outer regional and remote areas | Converted from the Accessibility and Remoteness Index of Australia (ARIA). Outer regional and remote areas were combined in to one category. |
| Number of bedrooms | Continuous | Number of bedrooms in the household of the recipient of care |
| Highest education level | 0. Year 11 and below 1. Bachelor and above 2. Certificates/ diploma 3. Year 12 | The highest education level for the recipient of care. The variable was originally asked only to people>=15, but we coded people <15 to category 0 as we assume that they are year 11 and below |
| Unemployment rate | Continuous variable | The mean unemployment rate periods: September 2014 to June 2015; September 2017 to June 2018. Each period is one year before the survey. |
