# Supplementary material for: Exploring the impact of a personalised disability reform on people with disability and their primary carers: Evidence from the Australian national disability insurance scheme
Source: PLoS One. 2025 May 7;20(5):e0321377. doi: 10.1371/journal.pone.0321377 (PMC12057950; doi:10.1371/journal.pone.0321377)
Supplement: S4 Table — (DOCX) [file pone.0321377.s004.docx]

### Table S4: Comparison of outcome and characteristics among the participants and non-participants observed in 2015 and 2018

|  | **(1) vs (2)** | **(3) vs (4)** | **(1) vs (3)** | **(2) vs (4)** |
| --- | --- | --- | --- | --- |
| **Outcomes (For primary carers)** |  |  |  |  |
| Weekly caring hours |  |  |  | 0.080* |
| Employed FT/PT ^ |  |  |  |  |
| Social participation without recipient |  |  |  |  |
| Any social participation |  |  |  |  |
| Total formal services (per week) |  |  |  |  |
| **Carer Characteristics** |  |  |  |  |
| Age | 0.075* |  | 0.038** | 0.099* |
| Male |  |  |  |  |
| Number of recipients of care | 0.085* |  |  | 0.098* |
| Adults (>=15yo) without disability in household | 0.035** |  | 0.052* |  |
| Highest education level N(%) |  |  |  |  |
| Year 11 and below |  |  |  |  |
| Bachelor and above |  |  |  |  |
| Certificates/diploma |  |  |  |  |
| Year 12 |  |  |  |  |
| **Recipient Characteristics** |  |  |  |  |
| Age |  |  |  |  |
| Male |  | 0.006** |  | 0.049** |
| Married/ De facto |  |  |  |  |
| Disability status (1. Profound; 0. Severe) | 0.070* |  | 0.040** |  |
| Born in Australia |  |  |  |  |
| Number of bedrooms in household | 0.077* | 0.026** |  |  |
| Highest education level N(%) |  |  |  |  |
| Year 11 and below |  |  |  |  |
| Bachelor and above |  |  |  |  |
| Certificates/diploma |  |  |  |  |
| Year 12 |  |  |  |  |
| Rurality (Aria+) N (%) | <0.001*** | 0.032** |  | 0.002** |
| Major cities |  |  |  |  |
| Inner regional |  |  |  |  |
| Outer regional and remote |  |  |  |  |
| Disability Type N (%) |  |  |  |  |
| Psychosocial |  |  |  |  |
| Other |  |  |  |  |

Notes: (1): Treatment group observed in wave 15; (2) Control group observed in wave 18; (3) Treatment group observed in wave 15; (4) Control group observed in wave 18
