# Supplementary material for: Exploring the impact of a personalised disability reform on people with disability and their primary carers: Evidence from the Australian national disability insurance scheme
Source: PLoS One. 2025 May 7;20(5):e0321377. doi: 10.1371/journal.pone.0321377 (PMC12057950; doi:10.1371/journal.pone.0321377)
Supplement: S5 Table — (DOCX) [file pone.0321377.s005.docx]

### Table S5: Main Analysis - complete table

|  | **(1)** | **(2)** | **(3)** | **(4)** | **(5)** | **(6)** | | **(7)** | |  |
| --- | --- | --- | --- | --- | --- | --- | --- | --- | --- | --- |
|  | **Formal services overall** | **Formal services extensive margin** | **Formal services intensive margin** | **Caring hours** | **Employment** | | **Social participation (Alone)** | | **Social participation (Any)** | |
| NDIS available area # Wave 18 | 0.0726 | -0.102 | -0.719 | 4.787 | -0.0530 | | -0.0668 | | -0.0966 | |
|  | (2.494) | (0.0739) | (4.315) | (3.853) | (0.0812) | | (0.0793) | | (0.0626) | |
| Wave 18 | -3.262* | 0.0388 | -5.430* | -3.289 | 0.0633 | | 0.0918 | | 0.0712 | |
|  | (1.807) | (0.0589) | (3.090) | (3.333) | (0.0708) | | (0.0674) | | (0.0614) | |
| **Carer Characteristics** |  |  |  |  |  | |  | |  | |
| Age of carer | -0.0498 | -0.0111 | 0.317 | 0.904** | 0.0222* | | -0.00338 | | 0.00296 | |
|  | (0.257) | (0.00997) | (0.488) | (0.360) | (0.0116) | | (0.00966) | | (0.00812) | |
| Age square of carer | 0.000420 | 0.000137 | -0.00325 | -0.00873** | -0.000271** | | -1.80e-05 | | -6.21e-05 | |
|  | (0.00282) | (0.000108) | (0.00521) | (0.00377) | (0.000132) | | (0.000107) | | (9.00e-05) | |
| Number of recipients of care | 1.405 | 0.0125 | 1.632 | 3.698*** | -0.0590** | | -0.0272 | | 0.00167 | |
|  | (1.605) | (0.0240) | (1.919) | (1.084) | (0.0272) | | (0.0241) | | (0.0251) | |
| Adults (>=15yo) without disability | -0.662 | -0.0145 | -1.470 | -1.109 | 0.0489** | | 0.0338* | | 0.0111 | |
|  | (0.589) | (0.0173) | (1.102) | (0.763) | (0.0203) | | (0.0197) | | (0.0138) | |
| Male | 2.430 | 0.00714 | 4.461 | -4.525** | 0.108** | | 0.0180 | | -0.0112 | |
|  | (1.947) | (0.0406) | (3.139) | (1.970) | (0.0523) | | (0.0427) | | (0.0320) | |
| Highest education: Bachelor and above | 0.890 | 0.0424 | -5.103 | -6.731*** | 0.360*** | | 0.206*** | | 0.205*** | |
|  | (1.572) | (0.0506) | (3.125) | (2.221) | (0.0560) | | (0.0460) | | (0.0326) | |
| Highest education: Certificates/diploma | 1.732* | 0.0768* | 0.506 | -2.296 | 0.181*** | | 0.0477 | | 0.0472 | |
|  | (0.974) | (0.0394) | (2.011) | (1.819) | (0.0436) | | (0.0448) | | (0.0415) | |
| Highest education: Year 12 | 1.193 | 0.128** | -2.246 | 0.212 | 0.0705 | | 0.115* | | 0.137*** | |
|  | (1.561) | (0.0550) | (3.235) | (2.253) | (0.0585) | | (0.0644) | | (0.0465) | |
| **Recipient Characteristics** |  |  |  |  |  | |  | |  | |
| Age | -0.505*** | -0.0160*** | -0.713*** | -0.803*** | 0.000703 | | 0.00404 | | -0.00456 | |
|  | (0.175) | (0.00448) | (0.219) | (0.173) | (0.00455) | | (0.00433) | | (0.00376) | |
| Age square | 0.00598*** | 0.000141** | 0.00885** | 0.0111*** | -1.81e-05 | | -1.09e-05 | | 5.02e-05 | |
|  | (0.00228) | (5.68e-05) | (0.00345) | (0.00232) | (6.02e-05) | | (5.54e-05) | | (4.98e-05) | |
| Number of bedrooms | 1.473* | 0.0232 | 4.073*** | -2.219** | 0.0381 | | 0.0485** | | 0.0224 | |
|  | (0.829) | (0.0208) | (1.547) | (0.857) | (0.0231) | | (0.0194) | | (0.0198) | |
| Male | 3.363** | 0.0109 | 7.171*** | 0.831 | -0.0617 | | -0.0381 | | -0.0907** | |
|  | (1.567) | (0.0405) | (2.206) | (1.683) | (0.0405) | | (0.0371) | | (0.0361) | |
| Married/De facto | -2.093* | -0.0735 | -3.689 | -7.025*** | 0.0565 | | -0.149*** | | -0.0508 | |
|  | (1.131) | (0.0511) | (2.351) | (2.091) | (0.0563) | | (0.0515) | | (0.0460) | |
| Highest education: Bachelor and above | 0.479 | 0.169** | -0.215 | -1.181 | 0.0754 | | 0.128** | | 0.152*** | |
|  | (2.907) | (0.0729) | (2.459) | (3.689) | (0.0683) | | (0.0570) | | (0.0460) | |
| Highest education: Certificates/diploma | -1.831 | 0.0680* | -1.020 | -3.023 | 0.0150 | | 0.115** | | 0.170*** | |
|  | (1.232) | (0.0397) | (2.805) | (2.218) | (0.0503) | | (0.0461) | | (0.0443) | |
| Highest education: Year 12 | -0.634 | 0.0568 | -0.179 | -1.793 | 0.109* | | 0.0397 | | 0.0283 | |
|  | (1.935) | (0.0532) | (3.525) | (2.791) | (0.0585) | | (0.0525) | | (0.0443) | |
| Born in Australia mainland | 3.723** | 0.0801 | 4.253* | -2.368 | 0.0155 | | 0.113** | | 0.109** | |
|  | (1.861) | (0.0509) | (2.251) | (2.338) | (0.0484) | | (0.0451) | | (0.0434) | |
| Profound disability | 5.442*** | 0.104 | 11.80** | 15.78*** | -0.0991 | | -0.140** | | -0.0194 | |
|  | (2.080) | (0.0903) | (4.658) | (2.714) | (0.0912) | | (0.0670) | | (0.0536) | |
| Rurality: Inner regional | -9.245*** | -0.180 | -24.74** | -2.908 | -0.0755 | | 0.121 | | 0.0559 | |
|  | (3.449) | (0.162) | (10.80) | (6.644) | (0.129) | | (0.0962) | | (0.0710) | |
| Rurality: Outer regional and remote | -13.30*** | -0.272 | -25.79* | -20.35*** | -0.0375 | | 0.228 | | -0.0137 | |
|  | (4.101) | (0.176) | (13.93) | (7.199) | (0.204) | | (0.152) | | (0.129) | |
| Psychosocial disability | 6.747*** | 0.0444 | 9.028** | 2.525 | -0.00922 | | -0.0103 | | 0.0493 | |
|  | (2.233) | (0.0422) | (3.483) | (2.040) | (0.0521) | | (0.0441) | | (0.0355) | |
| Unemployment rate | -1.513 | 0.00522 | -5.044* | 1.750 | 0.0320 | | 0.0105 | | 0.00771 | |
|  | (1.354) | (0.0301) | (3.015) | (1.491) | (0.0399) | | (0.0359) | | (0.0304) | |
| Constant | 12.05 | 0.764** | 22.55 | 10.27 | -0.315 | | 0.353 | | 0.581* | |
|  | (15.00) | (0.348) | (29.73) | (13.38) | (0.365) | | (0.317) | | (0.302) | |
| Observations | 1,052 | 1,052 | 511 | 1,052 | 939 | | 1,052 | | 1,052 | |
| R-squared | 0.132 | 0.122 | 0.197 | 0.165 | 0.142 | | 0.088 | | 0.114 | |
| Number of LGAs | 205 | 205 | 160 | 205 | 194 | | 205 | | 205 | |

Notes: Robust standard errors in parentheses, and they are clustered on the LGA-level; *** p<0.01, ** p<0.05, * p<0.1
