# Supplementary material for: Exploring the impact of a personalised disability reform on people with disability and their primary carers: Evidence from the Australian national disability insurance scheme
Source: PLoS One. 2025 May 7;20(5):e0321377. doi: 10.1371/journal.pone.0321377 (PMC12057950; doi:10.1371/journal.pone.0321377)
Supplement: S6 Table — (DOCX) [file pone.0321377.s006.docx]

### Table S6: The impact of NDIS on social participation of recipients

|  | **Social participation (Recipient)** |
| --- | --- |
| NDIS available area # Wave 18 | 0.101 |
|  | (0.115) |
| Wave 18 | -0.0831 |
|  | (0.104) |
| **Carer Characteristics** |  |
| Age of carer | -0.00873 |
|  | (0.0108) |
| Age square of carer | 7.13e-05 |
|  | (0.000118) |
| Number of recipients of care | 0.0726* |
|  | (0.0373) |
| Adults (>=15yo) without disability | 0.00208 |
|  | (0.0254) |
| Male | -0.0323 |
|  | (0.0617) |
| Highest education: Bachelor and above | 0.348*** |
|  | (0.0779) |
| Highest education: Certificates/diploma | 0.0830 |
|  | (0.0622) |
| Highest education: Year 12 | 0.259*** |
|  | -0.00873 |
| **Recipient Characteristics** |  |
| Age | -0.00729 |
|  | (0.00607) |
| Age square | 7.98e-05 |
|  | (8.77e-05) |
| Number of bedrooms | 0.0412 |
|  | (0.0366) |
| Male | -0.0634 |
|  | (0.0529) |
| Married/De facto | 0.0501 |
|  | (0.0792) |
| Highest education: Bachelor and above | 0.245*** |
|  | (0.0779) |
| Highest education: Certificates/diploma | 0.269*** |
|  | (0.0594) |
| Highest education: Year 12 | -0.0326 |
|  | (0.0881) |
| Born in Australia mainland | 0.166** |
|  | (0.0763) |
| Profound disability | 0.0456 |
|  | (0.0785) |
| Rurality: Inner regional | -0.230 |
|  | (0.201) |
| Rurality: Outer regional and remote | -0.141 |
|  | (0.300) |
| Psychosocial disability | 0.0704 |
|  | (0.0622) |
| Unemployment rate | 0.0682 |
|  | (0.0577) |
| Constant | 0.170 |
|  | (0.531) |
| Observations | 445 |
| Number of LGAs | 157 |
| R-squared | 0.192 |

Notes: Robust standard errors in parentheses, and they are clustered on the LGA-level; *** p<0.01, ** p<0.05, * p<0.1
