# Supplementary material for: Exploring the impact of a personalised disability reform on people with disability and their primary carers: Evidence from the Australian national disability insurance scheme
Source: PLoS One. 2025 May 7;20(5):e0321377. doi: 10.1371/journal.pone.0321377 (PMC12057950; doi:10.1371/journal.pone.0321377)
Supplement: S7 Table — (DOCX) [file pone.0321377.s007.docx]

### Table S7: Subgroup Analysis (15-24 vs other ages)

|  | **(1)** | **(2)** | **(3)** | **(4)** | **(5)** | **(6)** | **(7)** |
| --- | --- | --- | --- | --- | --- | --- | --- |
|  | **Formal services Overall** | **Formal services extensive margin** | **Formal services intensive margin** | **Caring hours** | **Employment** | **Social participation (Alone)** | **Social participation (Any)** |
| NDIS available area # Wave 18 | 2.074 | 0.154 | -0.157 | -0.103 | -0.354 | -6.833 | -2.847 |
|  | (11.07) | (0.160) | (0.173) | (0.177) | (0.241) | (11.28) | (7.534) |
| Wave 18 | -2.977 | 0.0906 | 0.0755 | 0.0705 | 0.0110 | -6.230** | -3.955** |
|  | (3.108) | (0.0768) | (0.0678) | (0.0682) | (0.0634) | (2.862) | (1.639) |
| NDIS available area #Wave 18 | 4.661 | -0.0717 | -0.0543 | -0.0835 | -0.0601 | -0.768 | 0.366 |
|  | (3.786) | (0.0853) | (0.0828) | (0.0711) | (0.0823) | (4.562) | (2.469) |
| Age 15-24 | -0.430 | 0.281** | 0.0309 | -0.00862 | -0.303*** | -9.527** | -8.290*** |
|  | (5.496) | (0.114) | (0.122) | (0.140) | (0.104) | (3.829) | (1.998) |
| NDIS available area# Age 15-24 | -4.643 | -0.176 | 0.0526 | 0.00388 | 0.299** | 5.266 | 6.171* |
|  | (6.888) | (0.146) | (0.158) | (0.152) | (0.126) | (5.129) | (3.158) |
| Wave 18# Age 15-24 | -2.520 | -0.246** | 0.163 | 0.0166 | 0.276 | 12.12 | 6.522 |
|  | (9.728) | (0.116) | (0.117) | (0.147) | (0.209) | (9.201) | (6.225) |
| **Carer Characteristics** |  |  |  |  |  |  |  |
| Age of carer | 1.007*** | 0.0214* | -0.00554 | 0.00385 | -0.00885 | 0.454 | -0.00195 |
|  | (0.358) | (0.0114) | (0.00969) | (0.00829) | (0.00995) | (0.477) | (0.251) |
| Age square of carer | -0.00965** | -0.000271** | 1.39e-06 | -7.05e-05 | 0.000116 | -0.00452 | 2.47e-05 |
|  | (0.00371) | (0.000129) | (0.000107) | (9.12e-05) | (0.000108) | (0.00507) | (0.00273) |
| Number of recipients of care | 3.706*** | -0.0597** | -0.0281 | 0.00106 | 0.0113 | 1.423 | 1.412 |
|  | (1.079) | (0.0271) | (0.0239) | (0.0253) | (0.0241) | (1.957) | (1.613) |
| Adults (>=15yo) without disability | -1.093 | 0.0481** | 0.0322* | 0.0114 | -0.0135 | -1.683 | -0.653 |
|  | (0.758) | (0.0202) | (0.0194) | (0.0138) | (0.0165) | (1.094) | (0.570) |
| Male | -4.831** | 0.116** | 0.0238 | -0.0132 | 0.00287 | 3.949 | 2.307 |
|  | (1.962) | (0.0538) | (0.0428) | (0.0320) | (0.0395) | (3.076) | (1.895) |
| Highest education: Bachelor and above | -6.959*** | 0.368*** | 0.210*** | 0.204*** | 0.0369 | -5.387* | 0.703 |
|  | (2.246) | (0.0541) | (0.0468) | (0.0323) | (0.0500) | (3.096) | (1.548) |
| Highest education: Certificates/diploma | -2.526 | 0.190*** | 0.0513 | 0.0457 | 0.0703* | 0.196 | 1.541 |
|  | (1.840) | (0.0433) | (0.0444) | (0.0419) | (0.0393) | (1.988) | (0.936) |
| Highest education: Year 12 | 0.0672 | 0.0776 | 0.118* | 0.138*** | 0.126** | -2.420 | 0.998 |
|  | (2.254) | (0.0589) | (0.0642) | (0.0469) | (0.0545) | (3.219) | (1.529) |
| **Recipient Characteristics** |  |  |  |  |  |  |  |
| Age | -0.763*** | -0.00100 | 0.00308 | -0.00402 | -0.0146*** | -0.700*** | -0.485*** |
|  | (0.178) | (0.00455) | (0.00446) | (0.00406) | (0.00455) | (0.211) | (0.170) |
| Age square | 0.0103*** | 1.17e-05 | 6.38e-06 | 4.12e-05 | 0.000118** | 0.00849** | 0.00561** |
|  | (0.00242) | (5.95e-05) | (5.78e-05) | (5.40e-05) | (5.88e-05) | (0.00363) | (0.00227) |
| Number of bedrooms | -2.196** | 0.0400* | 0.0486** | 0.0226 | 0.0215 | 4.163*** | 1.427* |
|  | (0.880) | (0.0230) | (0.0193) | (0.0199) | (0.0209) | (1.520) | (0.830) |
| Male | 0.649 | -0.0581 | -0.0351 | -0.0913** | 0.00877 | 6.876*** | 3.256** |
|  | (1.720) | (0.0397) | (0.0378) | (0.0367) | (0.0385) | (2.236) | (1.557) |
| Married/De facto | -7.527*** | 0.0750 | -0.137** | -0.0561 | -0.0893* | -4.210* | -2.381** |
|  | (2.007) | (0.0574) | (0.0527) | (0.0477) | (0.0502) | (2.361) | (1.098) |
| Highest education: Bachelor and above | -1.073 | 0.0741 | 0.127** | 0.150*** | 0.164** | 0.431 | 0.525 |
|  | (3.766) | (0.0679) | (0.0571) | (0.0465) | (0.0740) | (2.398) | (2.883) |
| Highest education: Certificates/diploma | -2.851 | 0.0140 | 0.113** | 0.170*** | 0.0647 | -0.569 | -1.790 |
|  | (2.252) | (0.0498) | (0.0472) | (0.0450) | (0.0401) | (2.822) | (1.247) |
| Highest education: Year 12 | -0.897 | 0.0865 | 0.0214 | 0.0312 | 0.0692 | 0.765 | -0.0925 |
|  | (2.764) | (0.0601) | (0.0522) | (0.0447) | (0.0532) | (3.491) | (1.730) |
| Born in Australia mainland | -2.408 | 0.0108 | 0.111** | 0.106** | 0.0819 | 4.235* | 3.909** |
|  | (2.311) | (0.0482) | (0.0452) | (0.0438) | (0.0506) | (2.222) | (1.854) |
| Profound disability | 16.07*** | -0.0975 | -0.146** | -0.0151 | 0.104 | 11.22** | 5.255** |
|  | (2.757) | (0.0896) | (0.0688) | (0.0539) | (0.0897) | (4.424) | (2.042) |
| Rurality: Inner regional | -2.501 | -0.0590 | 0.120 | 0.0649 | -0.187 | -25.39** | -9.860*** |
|  | (6.589) | (0.130) | (0.100) | (0.0715) | (0.153) | (10.68) | (3.506) |
| Rurality: Outer regional and remote | -20.02*** | 0.00922 | 0.231 | -0.00755 | -0.311* | -28.97* | -14.65*** |
|  | (6.992) | (0.211) | (0.158) | (0.128) | (0.163) | (14.96) | (4.282) |
| Psychosocial disability | 2.834 | -0.00625 | -0.0159 | 0.0523 | 0.0414 | 8.972** | 6.586*** |
|  | (2.091) | (0.0533) | (0.0453) | (0.0355) | (0.0426) | (3.593) | (2.310) |
| Unemployment rate | 1.735 | 0.0301 | 0.00932 | 0.00795 | 0.00836 | -4.951 | -1.454 |
|  | (1.473) | (0.0401) | (0.0362) | (0.0303) | (0.0304) | (3.036) | (1.352) |
| Constant | 7.957 | -0.303 | 0.415 | 0.553* | 0.710** | 21.09 | 11.54 |
|  | (13.15) | (0.365) | (0.316) | (0.303) | (0.348) | (29.01) | (14.81) |
| Observations | 1,052 | 939 | 1,052 | 1,052 | 1,052 | 511 | 1,052 |
| R-squared | 0.169 | 0.150 | 0.091 | 0.116 | 0.129 | 0.201 | 0.137 |
| Number of LGAs | 205 | 194 | 205 | 205 | 205 | 160 | 205 |

Notes: Robust standard errors in parentheses, and they are clustered on the LGA-level; *** p<0.01, ** p<0.05, * p<0.1
