# Supplementary material for: Exploring the impact of a personalised disability reform on people with disability and their primary carers: Evidence from the Australian national disability insurance scheme
Source: PLoS One. 2025 May 7;20(5):e0321377. doi: 10.1371/journal.pone.0321377 (PMC12057950; doi:10.1371/journal.pone.0321377)
Supplement: S8 Table — (DOCX) [file pone.0321377.s008.docx]

### Table S8: Sensitivity analysis: weighted sample

|  | **(1)** | **(2)** | **(3)** | **(4)** | **(5)** | **(6)** | **(7)** |
| --- | --- | --- | --- | --- | --- | --- | --- |
|  | **Formal services Overall** | **Formal services extensive margin** | **Formal services intensive margin** | **Caring hours** | **Employment** | **Social participation (Alone)** | **Social participation (Any)** |
| NDIS available area # Wave 18 | -0.469 | -0.157 | 0.260 | 1.977 | -0.022 | -0.012 | -0.060 |
|  | (0.830) | (0.084) | (0.950) | (0.616) | (0.846) | (0.898) | (0.416) |
| Observations | 1000 | 1000 | 1000 | 1000 | 1000 | 1000 | 1000 |
| Number of LGAs | 202 | 192 | 202 | 202 | 202 | 155 | 202 |

Notes: The weighted estimates and P-values were obtained used bootstrapping with 1,000 repetitions. Robust standard errors in parentheses; *** p<0.01, ** p<0.05, * p<0.1
