# Supplementary material for: Exploring the impact of a personalised disability reform on people with disability and their primary carers: Evidence from the Australian national disability insurance scheme
Source: PLoS One. 2025 May 7;20(5):e0321377. doi: 10.1371/journal.pone.0321377 (PMC12057950; doi:10.1371/journal.pone.0321377)
Supplement: S9 Table — (DOCX) [file pone.0321377.s009.docx]

### Table S9: Sensitivity analysis: Regional fixed effects

|  | **(1)** | **(2)** | **(3)** | **(4)** | **(5)** | **(6)** | **(7)** |
| --- | --- | --- | --- | --- | --- | --- | --- |
|  | **Formal services Overall** | **Formal services extensive margin** | **Formal services intensive margin** | **Caring hours** | **Employment** | **Social participation (Alone)** | **Social participation (Any)** |
| NDIS available area # Wave 18 | 0.989 | -0.0961 | 5.243 | 5.673 | -0.0857 | -0.0662 | -0.100 |
|  | (2.023) | (0.0719) | (4.020) | (3.759) | (0.0753) | (0.0766) | (0.0631) |
| Wave 18 | -2.321 | 0.0280 | -5.262 | -6.098* | 0.0860 | 0.0897 | 0.0643 |
|  | (1.766) | (0.0647) | (3.360) | (3.298) | (0.0703) | (0.0681) | (0.0579) |
| **Carer Characteristics** |  |  |  |  |  |  |  |
| Age of carer | -0.0135 | -0.0110 | 0.346 | 0.813** | 0.0221** | -0.00351 | 0.00302 |
|  | (0.250) | (0.00885) | (0.447) | (0.321) | (0.0105) | (0.00862) | (0.00666) |
| Age square of carer | 0.000397 | 0.000141 | -0.00354 | -0.00778** | -0.000261** | -1.97e-05 | -6.07e-05 |
|  | (0.00272) | (9.29e-05) | (0.00472) | (0.00331) | (0.000117) | (9.28e-05) | (7.05e-05) |
| Number of recipients of care | 0.952 | 0.0152 | 1.878 | 3.113*** | -0.0642*** | -0.0257 | 0.0119 |
|  | (1.750) | (0.0236) | (3.067) | (0.878) | (0.0240) | (0.0224) | (0.0202) |
| Adults (>=15yo) without disability | -0.321 | -0.00162 | -0.254 | -1.210 | 0.0483*** | 0.0371** | 0.0126 |
|  | (0.542) | (0.0183) | (0.948) | (0.732) | (0.0171) | (0.0181) | (0.0132) |
| Male | 2.263 | 0.0192 | 2.942 | -3.933** | 0.0944** | 0.0460 | 0.0136 |
|  | (1.741) | (0.0418) | (2.928) | (1.725) | (0.0400) | (0.0341) | (0.0325) |
| Highest education: Bachelor and above | 1.348 | 0.0478 | -0.540 | -6.421*** | 0.351*** | 0.234*** | 0.216*** |
|  | (1.396) | (0.0433) | (2.688) | (2.163) | (0.0530) | (0.0434) | (0.0313) |
| Highest education: Certificates/diploma | 2.316** | 0.0661* | 2.652 | -1.205 | 0.181*** | 0.0573 | 0.0574 |
|  | (1.033) | (0.0379) | (2.177) | (1.606) | (0.0398) | (0.0409) | (0.0392) |
| Highest education: Year 12 | 1.324 | 0.113** | -1.912 | 1.858 | 0.0772 | 0.0763 | 0.125** |
|  | (1.368) | (0.0465) | (3.152) | (1.614) | (0.0475) | (0.0607) | (0.0473) |
| **Recipient Characteristics** |  |  |  |  |  |  |  |
| Age | -0.414*** | -0.0128*** | -0.471** | -0.760*** | 0.000504 | 0.000782 | -0.00592* |
|  | (0.148) | (0.00419) | (0.189) | (0.176) | (0.00400) | (0.00403) | (0.00333) |
| Age square | 0.00477** | 9.67e-05* | 0.00638** | 0.0106*** | -2.12e-05 | 2.89e-05 | 5.96e-05 |
|  | (0.00182) | (5.40e-05) | (0.00260) | (0.00240) | (5.31e-05) | (5.61e-05) | (4.41e-05) |
| Number of bedrooms | 0.913 | 0.0171 | 1.339 | -1.562* | 0.0251 | 0.0259 | 0.00588 |
|  | (0.892) | (0.0187) | (2.062) | (0.793) | (0.0218) | (0.0202) | (0.0195) |
| Male | 2.960** | 0.00581 | 6.022** | 1.015 | -0.0535 | -0.00886 | -0.0710** |
|  | (1.478) | (0.0378) | (2.519) | (1.599) | (0.0366) | (0.0354) | (0.0323) |
| Married/De facto | -2.303** | -0.0719 | -4.652** | -7.386*** | 0.0721 | -0.124** | -0.0136 |
|  | (1.138) | (0.0504) | (2.173) | (2.021) | (0.0529) | (0.0529) | (0.0437) |
| Highest education: Bachelor and above | 0.196 | 0.156** | 0.872 | 0.255 | 0.0467 | 0.135** | 0.132*** |
|  | (2.103) | (0.0637) | (4.462) | (3.432) | (0.0642) | (0.0600) | (0.0462) |
| Highest education: Certificates/diploma | -1.867* | 0.0440 | -2.835 | -2.498 | -0.0162 | 0.101** | 0.141*** |
|  | (1.103) | (0.0305) | (3.056) | (2.226) | (0.0446) | (0.0397) | (0.0401) |
| Highest education: Year 12 | -0.456 | 0.0533 | -0.384 | -0.179 | 0.0743 | 0.0551 | 0.0343 |
|  | (2.086) | (0.0479) | (3.495) | (3.085) | (0.0516) | (0.0457) | (0.0385) |
| Born in Australia mainland | 4.161*** | 0.0954** | 6.594** | -2.160 | 0.0257 | 0.116*** | 0.100** |
|  | (1.543) | (0.0413) | (2.511) | (2.145) | (0.0491) | (0.0358) | (0.0390) |
| Profound disability | 4.724* | 0.0799 | 9.893 | 13.06*** | -0.0989 | -0.142** | -0.0362 |
|  | (2.573) | (0.0880) | (6.808) | (2.584) | (0.0847) | (0.0568) | (0.0453) |
| Rurality: Inner regional | -7.335*** | -0.0640 | -13.64*** | -8.175* | -0.00697 | 0.0497 | 0.106** |
|  | (2.304) | (0.0718) | (4.259) | (4.538) | (0.0720) | (0.0588) | (0.0501) |
| Rurality: Outer regional and remote | -12.80*** | -0.256** | -20.09*** | -15.10*** | -0.0510 | -0.0491 | -0.0288 |
|  | (2.647) | (0.117) | (5.164) | (5.286) | (0.102) | (0.0937) | (0.0785) |
| Psychosocial disability | 6.300*** | 0.0172 | 8.499*** | 2.677 | -0.0353 | 0.0151 | 0.0610* |
|  | (2.040) | (0.0366) | (3.003) | (1.811) | (0.0480) | (0.0449) | (0.0322) |
| Unemployment rate | -0.180 | 0.00375 | -0.438 | 0.289 | -0.0198** | -0.00248 | -0.00332 |
|  | (0.296) | (0.0137) | (0.613) | (0.368) | (0.00950) | (0.00873) | (0.00681) |
| Constant | 2.086 | 0.716** | -8.778 | 22.23** | 0.0481 | 0.572** | 0.698*** |
|  | (10.17) | (0.289) | (19.53) | (8.977) | (0.244) | (0.242) | (0.194) |
| Observations | 1,052 | 1,052 | 511 | 1,052 | 939 | 1,052 | 1,052 |
| R-squared | 0.121 | 0.113 | 0.148 | 0.141 | 0.141 | 0.087 | 0.114 |
| Number of LGAs | 60 | 60 | 58 | 60 | 60 | 60 | 60 |

Notes: Robust standard errors in parentheses, and they are clustered on the LGA-level; *** p<0.01, ** p<0.05, * p<0.1
