# Supplementary material for: Exploring the impact of a personalised disability reform on people with disability and their primary carers: Evidence from the Australian national disability insurance scheme
Source: PLoS One. 2025 May 7;20(5):e0321377. doi: 10.1371/journal.pone.0321377 (PMC12057950; doi:10.1371/journal.pone.0321377)
Supplement: S10 Table — (DOCX) [file pone.0321377.s010.docx]

### Table S10: Sensitivity analysis: Cut-off date at 30 June 2017

|  | **(1)** | **(2)** | **(3)** | **(4)** | **(5)** | **(6)** | **(7)** |
| --- | --- | --- | --- | --- | --- | --- | --- |
|  | **Formal services Overall** | **Formal services extensive margin** | **Formal services intensive margin** | **Caring hours** | **Employment** | **Social participation (Alone)** | **Social participation (Any)** |
| NDIS available area # Wave 18 | 0.352 | -0.0288 | -3.910 | 1.894 | -0.0881 | -0.0330 | -0.135* |
|  | (3.864) | (0.0819) | (6.782) | (4.290) | (0.0975) | (0.108) | (0.0709) |
| Wave 18 | -3.178* | 0.0445 | -4.489 | -3.037 | 0.0588 | 0.0903 | 0.0747 |
|  | (1.802) | (0.0580) | (3.768) | (3.327) | (0.0744) | (0.0706) | (0.0650) |
| **Carer Characteristics** |  |  |  |  |  |  |  |
| Age of carer | -0.348 | -0.000554 | 0.270 | 0.529 | 0.0263* | -0.00332 | -0.00113 |
|  | (0.382) | (0.0159) | (0.708) | (0.480) | (0.0154) | (0.0158) | (0.0123) |
| Age square of carer | 0.00420 | 1.01e-05 | -0.00200 | -0.00488 | -0.000328* | -6.70e-06 | 5.44e-06 |
|  | (0.00417) | (0.000171) | (0.00740) | (0.00477) | (0.000178) | (0.000179) | (0.000135) |
| Number of recipients of care | -0.663 | 0.00594 | 0.142 | 2.119 | -0.0709** | -0.000175 | 0.000282 |
|  | (1.297) | (0.0284) | (2.806) | (1.500) | (0.0342) | (0.0296) | (0.0304) |
| Adults (>=15yo) without disability | -0.380 | -0.0116 | -0.103 | -1.531 | 0.0397 | 0.0354 | 0.0127 |
|  | (0.838) | (0.0266) | (1.822) | (1.000) | (0.0252) | (0.0263) | (0.0188) |
| Male | 5.157* | 0.0760 | 6.914 | -3.853 | 0.113* | 0.0343 | 0.0143 |
|  | (2.731) | (0.0537) | (4.916) | (2.404) | (0.0679) | (0.0599) | (0.0442) |
| Highest education: Bachelor and above | 1.290 | 0.0445 | -3.500 | -4.376 | 0.285*** | 0.224*** | 0.225*** |
|  | (2.076) | (0.0639) | (4.184) | (2.870) | (0.0691) | (0.0562) | (0.0390) |
| Highest education: Certificates/diploma | 3.070* | 0.0753 | 1.039 | -0.193 | 0.121** | 0.0513 | 0.0897 |
|  | (1.683) | (0.0505) | (3.311) | (2.422) | (0.0541) | (0.0622) | (0.0541) |
| Highest education: Year 12 | 2.707 | 0.110 | 0.920 | -1.009 | 0.0190 | 0.135 | 0.154** |
|  | (2.227) | (0.0773) | (4.730) | (3.225) | (0.0826) | (0.0871) | (0.0648) |
| **Recipient Characteristics** |  |  |  |  |  |  |  |
| Age | -0.493* | -0.0156** | -0.741** | -0.406* | 0.00196 | -0.00184 | -0.00731 |
|  | (0.266) | (0.00628) | (0.338) | (0.228) | (0.00604) | (0.00546) | (0.00496) |
| Age square | 0.00524* | 0.000135* | 0.0102* | 0.00604** | -3.36e-05 | 4.76e-05 | 6.67e-05 |
|  | (0.00309) | (7.78e-05) | (0.00549) | (0.00294) | (7.99e-05) | (6.90e-05) | (6.18e-05) |
| Number of bedrooms | 2.209** | 0.0529** | 4.061* | -2.821** | 0.0540* | 0.0718*** | 0.0312 |
|  | (0.920) | (0.0264) | (2.054) | (1.152) | (0.0310) | (0.0263) | (0.0257) |
| Male | 6.271*** | 0.0215 | 9.234*** | 1.544 | -0.0388 | -0.0525 | -0.0477 |
|  | (2.025) | (0.0561) | (3.238) | (2.182) | (0.0534) | (0.0467) | (0.0518) |
| Married/De facto | -2.054 | -0.130** | -3.765 | -13.17*** | 0.0620 | -0.0752 | 0.0128 |
|  | (1.944) | (0.0654) | (2.843) | (2.351) | (0.0703) | (0.0640) | (0.0663) |
| Highest education: Bachelor and above | -1.825 | 0.143 | -1.699 | 1.495 | 0.0556 | 0.164** | 0.137** |
|  | (2.389) | (0.0977) | (4.969) | (5.507) | (0.0935) | (0.0733) | (0.0553) |
| Highest education: Certificates/diploma | -2.280 | 0.108* | -3.300 | -1.191 | 0.0339 | 0.157** | 0.159** |
|  | (1.989) | (0.0647) | (5.292) | (3.305) | (0.0691) | (0.0625) | (0.0623) |
| Highest education: Year 12 | -5.684** | 0.105 | -7.500** | -3.317 | 0.0966 | 0.0652 | -0.0157 |
|  | (2.313) | (0.0735) | (3.464) | (4.113) | (0.0712) | (0.0788) | (0.0620) |
| Born in Australia mainland | 2.398 | 0.0501 | 7.761** | -5.610* | 0.101* | 0.118* | 0.0639 |
|  | (1.476) | (0.0641) | (3.222) | (3.016) | (0.0549) | (0.0641) | (0.0622) |
| Profound disability | 5.597** | 0.153 | 17.92** | 15.18*** | -0.0556 | -0.0338 | 0.0282 |
|  | (2.692) | (0.127) | (6.859) | (4.004) | (0.126) | (0.0967) | (0.0752) |
| Rurality: Inner regional | -12.58*** | -0.129 | -27.61** | 0.624 | -0.0608 | 0.114 | 0.0803 |
|  | (4.111) | (0.197) | (10.62) | (7.624) | (0.138) | (0.0950) | (0.0615) |
| Rurality: Outer regional and remote | -17.99*** | -0.202 | -32.13** | -15.53** | -0.114 | 0.292 | -0.114 |
|  | (5.212) | (0.212) | (14.19) | (7.721) | (0.219) | (0.177) | (0.138) |
| Psychosocial disability | 5.820* | -0.0293 | 8.131 | 2.962 | -0.00251 | 0.00397 | 0.0456 |
|  | (3.001) | (0.0532) | (5.517) | (2.632) | (0.0684) | (0.0527) | (0.0493) |
| Unemployment rate | -0.237 | 0.0271 | -2.786 | 1.623 | 0.0197 | 0.0376 | 0.0431 |
|  | (2.198) | (0.0371) | (5.172) | (2.295) | (0.0537) | (0.0521) | (0.0466) |
| Constant | 10.54 | 0.269 | -0.0384 | 23.45 | -0.419 | -0.0427 | 0.347 |
|  | (21.27) | (0.460) | (46.57) | (17.83) | (0.495) | (0.421) | (0.458) |
| Observations | 602 | 602 | 304 | 602 | 545 | 602 | 602 |
| R-squared | 0.157 | 0.136 | 0.211 | 0.166 | 0.128 | 0.101 | 0.116 |
| Number of LGAs | 98 | 98 | 81 | 98 | 93 | 98 | 98 |

Notes: Robust standard errors in parentheses, and they are clustered on the LGA-level; *** p<0.01, ** p<0.05, * p<0.1
