# Supplementary material for: Exploring the impact of a personalised disability reform on people with disability and their primary carers: Evidence from the Australian national disability insurance scheme
Source: PLoS One. 2025 May 7;20(5):e0321377. doi: 10.1371/journal.pone.0321377 (PMC12057950; doi:10.1371/journal.pone.0321377)
Supplement: S11 Table — (DOCX) [file pone.0321377.s011.docx]

### Table S11: Sensitivity analysis: Main recipients only

|  | **(1)** | **(2)** | **(3)** | **(4)** | **(5)** | **(6)** | **(7)** |
| --- | --- | --- | --- | --- | --- | --- | --- |
|  | **Formal services Overall** | **Formal services extensive margin** | **Formal services intensive margin** | **Caring hours** | **Employment** | **Social participation (Alone)** | **Social participation (Any)** |
| NDIS available area # Wave 18 | -0.756 | -0.0744 | -4.833 | 4.804 | -0.111 | -0.108 | -0.115* |
|  | (2.638) | (0.0768) | (4.993) | (4.229) | (0.0831) | (0.0841) | (0.0646) |
| Wave 18 | -2.553 | 0.0273 | -3.058 | -3.354 | 0.0963 | 0.103 | 0.0740 |
|  | (1.805) | (0.0630) | (3.842) | (3.725) | (0.0723) | (0.0698) | (0.0636) |
| **Carer Characteristics** |  |  |  |  |  |  |  |
| Age of carer | -0.0414 | -0.0107 | 0.272 | 0.890** | 0.0218* | -0.00292 | 0.00343 |
|  | (0.273) | (0.00981) | (0.531) | (0.381) | (0.0116) | (0.00951) | (0.00840) |
| Age square of carer | 0.000226 | 0.000132 | -0.00284 | -0.00833** | -0.000252* | -2.73e-05 | -6.99e-05 |
|  | (0.00301) | (0.000106) | (0.00576) | (0.00396) | (0.000132) | (0.000106) | (9.24e-05) |
| Number of recipients of care | 2.547 | 0.00886 | 2.745 | 3.538*** | -0.0421 | -0.0308 | 0.00949 |
|  | (1.894) | (0.0259) | (2.057) | (1.175) | (0.0304) | (0.0264) | (0.0264) |
| Adults (>=15yo) without disability | -0.590 | -0.0257 | -1.171 | -0.861 | 0.0644*** | 0.0352* | 0.0122 |
|  | (0.638) | (0.0185) | (1.162) | (0.858) | (0.0215) | (0.0202) | (0.0140) |
| Male | 2.931 | 0.0360 | 4.267 | -5.269** | 0.113** | 0.0271 | 0.0108 |
|  | (2.121) | (0.0444) | (3.317) | (2.111) | (0.0534) | (0.0435) | (0.0346) |
| Highest education: Bachelor and above | 0.577 | 0.0358 | -6.548* | -7.244*** | 0.364*** | 0.207*** | 0.224*** |
|  | (1.616) | (0.0517) | (3.681) | (2.282) | (0.0597) | (0.0482) | (0.0362) |
| Highest education: Certificates/diploma | 1.389 | 0.0757* | -1.027 | -2.633 | 0.179*** | 0.0545 | 0.0687 |
|  | (1.007) | (0.0421) | (2.445) | (1.953) | (0.0462) | (0.0453) | (0.0442) |
| Highest education: Year 12 | 0.533 | 0.116** | -4.197 | 0.218 | 0.0732 | 0.101 | 0.132*** |
|  | (1.628) | (0.0552) | (3.671) | (2.350) | (0.0598) | (0.0657) | (0.0476) |
| **Recipient Characteristics** |  |  |  |  |  |  |  |
| Age | -0.494*** | -0.0164*** | -0.723*** | -0.716*** | -0.000946 | 0.00617 | -0.00316 |
|  | (0.188) | (0.00457) | (0.252) | (0.186) | (0.00485) | (0.00446) | (0.00384) |
| Age square | 0.00593** | 0.000138** | 0.00966** | 0.00952*** | 2.33e-06 | -3.10e-05 | 3.78e-05 |
|  | (0.00248) | (5.83e-05) | (0.00385) | (0.00248) | (6.48e-05) | (5.68e-05) | (4.99e-05) |
| Number of bedrooms | 1.208 | 0.0164 | 4.358** | -2.307** | 0.0315 | 0.0500** | 0.0184 |
|  | (0.874) | (0.0212) | (1.699) | (0.930) | (0.0255) | (0.0201) | (0.0195) |
| Male | 4.288** | 0.0331 | 7.657*** | 0.300 | -0.0879** | -0.0334 | -0.0638* |
|  | (1.691) | (0.0436) | (2.287) | (1.812) | (0.0427) | (0.0362) | (0.0383) |
| Married/De facto | -2.459** | -0.0781 | -4.824* | -6.313*** | 0.0415 | -0.170*** | -0.0695 |
|  | (1.134) | (0.0530) | (2.656) | (2.197) | (0.0560) | (0.0518) | (0.0472) |
| Highest education: Bachelor and above | 0.989 | 0.177** | 0.742 | -1.099 | 0.0639 | 0.114* | 0.126** |
|  | (3.109) | (0.0696) | (2.396) | (4.096) | (0.0750) | (0.0610) | (0.0523) |
| Highest education: Certificates/diploma | -1.444 | 0.0792* | -0.470 | -2.789 | 0.0138 | 0.0990** | 0.158*** |
|  | (1.210) | (0.0418) | (2.860) | (2.284) | (0.0528) | (0.0488) | (0.0445) |
| Highest education: Year 12 | -0.750 | 0.0445 | 0.136 | -2.443 | 0.111** | 0.0366 | 0.0333 |
|  | (2.087) | (0.0551) | (3.810) | (2.864) | (0.0544) | (0.0585) | (0.0488) |
| Born in Australia mainland | 3.785* | 0.0839 | 4.469* | -2.048 | 0.0159 | 0.121** | 0.103** |
|  | (2.014) | (0.0512) | (2.521) | (2.382) | (0.0518) | (0.0470) | (0.0426) |
| Profound disability | 6.418*** | 0.132 | 11.98* | 12.09*** | -0.166 | -0.106 | 0.0372 |
|  | (2.363) | (0.115) | (6.304) | (3.578) | (0.114) | (0.0862) | (0.0707) |
| Rurality: Inner regional | -10.50*** | -0.156 | -26.92** | -2.985 | -0.0765 | 0.145 | 0.0541 |
|  | (3.630) | (0.171) | (11.64) | (7.507) | (0.133) | (0.106) | (0.0828) |
| Rurality: Outer regional and remote | -14.40*** | -0.212 | -30.15** | -21.69*** | -0.0151 | 0.282* | 0.0176 |
|  | (4.258) | (0.183) | (14.85) | (8.017) | (0.209) | (0.170) | (0.155) |
| Psychosocial disability | 7.044*** | 0.0456 | 10.13** | 2.888 | -0.000229 | -0.0187 | 0.0468 |
|  | (2.496) | (0.0453) | (4.109) | (2.189) | (0.0541) | (0.0467) | (0.0376) |
| Unemployment rate | -1.411 | 0.0144 | -6.124 | 1.318 | 0.0305 | -0.00394 | 0.00214 |
|  | (1.553) | (0.0338) | (3.939) | (1.734) | (0.0432) | (0.0399) | (0.0343) |
| Constant | 9.730 | 0.686* | 29.00 | 16.49 | -0.239 | 0.360 | 0.515 |
|  | (15.77) | (0.368) | (34.29) | (14.98) | (0.382) | (0.340) | (0.323) |
| Observations | 978 | 978 | 469 | 978 | 871 | 978 | 978 |
| R-squared | 0.140 | 0.129 | 0.213 | 0.147 | 0.145 | 0.089 | 0.108 |
| Number of LGAs | 202 | 202 | 152 | 202 | 192 | 202 | 202 |

Notes: Robust standard errors in parentheses, and they are clustered on the LGA-level; *** p<0.01, ** p<0.05, * p<0.1
