# Supplementary material for: Exploring the impact of a personalised disability reform on people with disability and their primary carers: Evidence from the Australian national disability insurance scheme
Source: PLoS One. 2025 May 7;20(5):e0321377. doi: 10.1371/journal.pone.0321377 (PMC12057950; doi:10.1371/journal.pone.0321377)
Supplement: S12 Table — (DOCX) [file pone.0321377.s012.docx]

### Table S12: Sensitivity Analysis: Excluding carers eligible for NDIS

|  | **(1)** | **(2)** | **(3)** | **(4)** | **(5)** | **(6)** | **(7)** |
| --- | --- | --- | --- | --- | --- | --- | --- |
|  | **Formal services Overall** | **Formal services extensive margin** | **Formal services intensive margin** | **Caring hours** | **Employment** | **Social participation (Alone)** | **Social participation (Any)** |
| NDIS available area # Wave 18 | -0.295 | -0.0935 | -0.975 | 4.295 | -0.0465 | -0.0745 | -0.101 |
|  | (2.669) | (0.0762) | (5.050) | (3.963) | (0.0822) | (0.0816) | (0.0654) |
| Wave 18 | -3.553* | 0.0382 | -5.693* | -3.168 | 0.0596 | 0.104 | 0.0781 |
|  | (1.817) | (0.0585) | (3.142) | (3.351) | (0.0696) | (0.0681) | (0.0628) |
| **Carer Characteristics** |  |  |  |  |  |  |  |
| Age of carer | 0.00301 | -0.0103 | 0.389 | 0.879** | 0.0243* | -0.00393 | 0.00116 |
|  | (0.259) | (0.0109) | (0.491) | (0.376) | (0.0125) | (0.00963) | (0.00841) |
| Age square of carer | -7.35e-05 | 0.000126 | -0.00401 | -0.00880** | -0.000288** | -3.19e-06 | -3.83e-05 |
|  | (0.00284) | (0.000117) | (0.00530) | (0.00392) | (0.000140) | (0.000107) | (9.14e-05) |
| Number of recipients of care | 2.191 | 0.00127 | 3.054 | 2.952** | -0.0635** | -0.0191 | -0.00970 |
|  | (1.806) | (0.0257) | (2.368) | (1.256) | (0.0318) | (0.0254) | (0.0223) |
| Adults (>=15yo) without disability | -0.871 | -0.00899 | -1.884 | -1.114 | 0.0376* | 0.0458** | 0.0228 |
|  | (0.641) | (0.0190) | (1.317) | (0.865) | (0.0201) | (0.0207) | (0.0157) |
| Male | 3.346 | 0.0246 | 6.050* | -2.771 | 0.0957 | 0.0249 | 0.0187 |
|  | (2.085) | (0.0438) | (3.418) | (1.928) | (0.0579) | (0.0457) | (0.0341) |
| Highest education: Bachelor and above | 0.715 | 0.0692 | -6.433 | -5.570** | 0.340*** | 0.206*** | 0.212*** |
|  | (1.878) | (0.0512) | (4.214) | (2.246) | (0.0616) | (0.0500) | (0.0374) |
| Highest education: Certificates/diploma | 1.168 | 0.0787* | -1.099 | -1.506 | 0.176*** | 0.0471 | 0.0448 |
|  | (1.146) | (0.0422) | (2.396) | (1.893) | (0.0514) | (0.0487) | (0.0479) |
| Highest education: Year 12 | 0.358 | 0.122** | -4.084 | 0.782 | 0.0589 | 0.143** | 0.124** |
|  | (1.785) | (0.0572) | (3.713) | (2.568) | (0.0596) | (0.0695) | (0.0507) |
| **Recipient Characteristics** |  |  |  |  |  |  |  |
| Age | -0.523*** | -0.0152*** | -0.751*** | -0.622*** | -0.00174 | 0.00500 | -0.00359 |
|  | (0.189) | (0.00484) | (0.230) | (0.182) | (0.00494) | (0.00438) | (0.00400) |
| Age square | 0.00572** | 0.000129** | 0.00823** | 0.00837*** | 1.29e-05 | -2.13e-05 | 3.60e-05 |
|  | (0.00239) | (5.97e-05) | (0.00342) | (0.00243) | (6.41e-05) | (5.59e-05) | (5.23e-05) |
| Number of bedrooms | 1.470* | 0.0221 | 4.764*** | -1.970** | 0.0582** | 0.0413** | 0.00286 |
|  | (0.886) | (0.0230) | (1.745) | (0.923) | (0.0257) | (0.0202) | (0.0191) |
| Male | 4.243** | 0.00486 | 8.546*** | 1.419 | -0.0665 | -0.0494 | -0.0691* |
|  | (1.637) | (0.0454) | (2.501) | (1.752) | (0.0438) | (0.0406) | (0.0395) |
| Married/De facto | -1.440 | -0.0822 | -1.151 | -5.860** | 0.0504 | -0.172*** | -0.0784 |
|  | (1.189) | (0.0556) | (2.058) | (2.358) | (0.0613) | (0.0539) | (0.0515) |
| Highest education: Bachelor and above | 1.495 | 0.155** | 1.540 | -3.007 | 0.0874 | 0.138** | 0.141*** |
|  | (2.946) | (0.0758) | (2.892) | (4.008) | (0.0732) | (0.0567) | (0.0507) |
| Highest education: Certificates/diploma | -1.515 | 0.0734 | -1.352 | -4.621* | 0.0369 | 0.121** | 0.174*** |
|  | (1.253) | (0.0456) | (2.980) | (2.485) | (0.0570) | (0.0484) | (0.0483) |
| Highest education: Year 12 | -0.403 | 0.0613 | 0.244 | -2.464 | 0.114* | 0.0509 | 0.0233 |
|  | (2.033) | (0.0567) | (3.460) | (2.897) | (0.0625) | (0.0578) | (0.0477) |
| Born in Australia mainland | 3.121* | 0.0823 | 4.553* | -2.319 | 0.0149 | 0.109** | 0.0979** |
|  | (1.751) | (0.0503) | (2.379) | (2.405) | (0.0521) | (0.0460) | (0.0460) |
| Profound disability | 5.214** | 0.110 | 8.150 | 15.57*** | -0.0971 | -0.150** | -0.00280 |
|  | (2.065) | (0.0970) | (5.608) | (2.872) | (0.0992) | (0.0733) | (0.0603) |
| Rurality: Inner regional | -9.218** | -0.140 | -25.99** | -0.498 | -0.0996 | 0.0853 | 0.0757 |
|  | (3.557) | (0.171) | (10.60) | (6.677) | (0.135) | (0.0932) | (0.0750) |
| Rurality: Outer regional and remote | -12.07*** | -0.182 | -27.89** | -20.47*** | 0.0624 | 0.215 | -0.00970 |
|  | (4.511) | (0.192) | (14.04) | (7.206) | (0.212) | (0.166) | (0.156) |
| Psychosocial disability | 8.150*** | 0.0526 | 9.495** | 3.039 | -0.00270 | -0.0303 | 0.0527 |
|  | (2.540) | (0.0468) | (3.861) | (2.155) | (0.0571) | (0.0484) | (0.0373) |
| Unemployment rate | -1.660 | 0.00428 | -5.546 | 1.957 | 0.0301 | 0.0315 | 0.0174 |
|  | (1.542) | (0.0311) | (3.461) | (1.583) | (0.0388) | (0.0375) | (0.0325) |
| Constant | 11.95 | 0.713** | 25.99 | 7.011 | -0.368 | 0.215 | 0.576* |
|  | (16.15) | (0.352) | (32.56) | (13.68) | (0.365) | (0.325) | (0.318) |
| Observations | 933 | 933 | 447 | 933 | 821 | 933 | 933 |
| R-squared | 0.146 | 0.123 | 0.225 | 0.146 | 0.143 | 0.096 | 0.117 |
| Number of LGAs | 198 | 198 | 150 | 198 | 185 | 198 | 198 |

Notes: Robust standard errors in parentheses, and they are clustered on the LGA-level; *** p<0.01, ** p<0.05, * p<0.1
