# Supplementary material for: Exploring the impact of a personalised disability reform on people with disability and their primary carers: Evidence from the Australian national disability insurance scheme
Source: PLoS One. 2025 May 7;20(5):e0321377. doi: 10.1371/journal.pone.0321377 (PMC12057950; doi:10.1371/journal.pone.0321377)
Supplement: S13 Table — (DOCX) [file pone.0321377.s013.docx]

### Table S13: Sensitivity Analysis—Different control group (not eligible and live in NDIS available areas)

|  | **(1)** | **(2)** | **(3)** | **(4)** | **(5)** | **(6)** | **(7)** |
| --- | --- | --- | --- | --- | --- | --- | --- |
|  | **Formal services Overall** | **Formal services extensive margin** | **Formal services intensive margin** | **Caring hours** | **Employment** | **Social participation (Alone)** | **Social participation (Any)** |
| NDIS available area # Wave 18 | 0.165 | -0.0692 | 2.345 | 1.045 | 0.0697 | 0.0667 | 0.0121 |
|  | (1.737) | (0.0562) | (3.349) | (2.555) | (0.0821) | (0.0553) | (0.0422) |
| Wave 18 | -2.009** | 0.0106 | -4.253* | -0.234 | -0.0242 | -0.0389 | -0.0434 |
|  | (0.925) | (0.0453) | (2.177) | (1.934) | (0.0724) | (0.0414) | (0.0331) |
| **Carer Characteristics** |  |  |  |  |  |  |  |
| Age of carer | 0.139 | -0.00955 | 0.372 | 0.686*** | 0.0274** | -0.00476 | -0.00302 |
|  | (0.149) | (0.00610) | (0.271) | (0.238) | (0.0122) | (0.00478) | (0.00414) |
| Age square of carer | -0.00114 | 0.000116** | -0.00389* | -0.0058*** | -0.000327** | 1.67e-07 | 1.84e-06 |
|  | (0.00124) | (5.53e-05) | (0.00224) | (0.00221) | (0.000135) | (4.52e-05) | (3.95e-05) |
| Number of recipients of care | 0.898 | 0.0169 | -0.578 | 4.532*** | -0.0329 | -0.0350 | -0.0175 |
|  | (1.241) | (0.0243) | (1.123) | (0.980) | (0.0261) | (0.0236) | (0.0230) |
| Adults (>=15yo) without disability | -0.505 | -0.0434*** | -0.878 | -0.220 | 0.0526** | 0.0117 | -0.00121 |
|  | (0.420) | (0.0144) | (0.860) | (0.590) | (0.0233) | (0.0156) | (0.0133) |
| Male | -0.348 | 0.00869 | 0.826 | -2.997* | 0.00797 | -0.0176 | -0.0261 |
|  | (0.894) | (0.0345) | (1.683) | (1.586) | (0.0458) | (0.0341) | (0.0264) |
| Highest education: Bachelor and above | -0.262 | 0.0794* | -4.094** | -6.750*** | 0.437*** | 0.169*** | 0.174*** |
|  | (0.835) | (0.0409) | (1.913) | (1.857) | (0.0532) | (0.0387) | (0.0270) |
| Highest education: Certificates/diploma | 0.608 | 0.0380 | 0.507 | -3.654** | 0.223*** | 0.104*** | 0.0609** |
|  | (0.650) | (0.0348) | (1.858) | (1.503) | (0.0470) | (0.0282) | (0.0245) |
| Highest education: Year 12 | -0.658 | 0.119*** | -5.429** | -0.724 | 0.231*** | 0.0845* | 0.125*** |
|  | (1.092) | (0.0411) | (2.598) | (1.746) | (0.0527) | (0.0502) | (0.0360) |
| **Recipient Characteristics** |  |  |  |  |  |  |  |
| Age | -0.227*** | -0.0118*** | -0.214 | -0.648*** | 0.000669 | 0.00307 | -0.00549** |
|  | (0.0832) | (0.00259) | (0.156) | (0.115) | (0.00393) | (0.00288) | (0.00242) |
| Age square | 0.00202*** | 9.64e-05*** | 0.00230* | 0.00769*** | 3.74e-07 | -2.11e-05 | 3.76e-05 |
|  | (0.000737) | (3.00e-05) | (0.00128) | (0.00118) | (4.07e-05) | (3.02e-05) | (2.60e-05) |
| Number of bedrooms | 0.438 | 0.00670 | 2.017* | -1.740** | 0.00933 | 0.0215 | 0.0304** |
|  | (0.543) | (0.0155) | (1.062) | (0.865) | (0.0207) | (0.0167) | (0.0148) |
| Male | 0.498 | 0.00827 | 2.143 | 1.242 | -0.124*** | -0.0221 | -0.0935*** |
|  | (0.931) | (0.0301) | (1.593) | (1.592) | (0.0387) | (0.0330) | (0.0247) |
| Married/De facto | -1.518* | -0.0902** | -2.318 | -3.200* | 0.0544 | -0.0432 | 0.0378 |
|  | (0.912) | (0.0376) | (1.568) | (1.846) | (0.0587) | (0.0431) | (0.0406) |
| Highest education: Bachelor and above | 0.363 | 0.119** | -1.812 | -1.156 | 0.0685 | 0.0787* | 0.125*** |
|  | (1.458) | (0.0532) | (1.987) | (2.515) | (0.0591) | (0.0469) | (0.0348) |
| Highest education: Certificates/diploma | -1.333 | 0.0192 | -1.904 | -1.659 | -0.00217 | 0.0703** | 0.130*** |
|  | (0.817) | (0.0353) | (1.716) | (1.716) | (0.0535) | (0.0299) | (0.0267) |
| Highest education: Year 12 | 1.144 | 0.00621 | 2.021 | 1.946 | 0.0232 | 0.0237 | 0.0418 |
|  | (1.465) | (0.0451) | (3.062) | (2.253) | (0.0669) | (0.0474) | (0.0456) |
| Born in Australia mainland | 1.962* | 0.0724* | 1.094 | -2.613* | 0.0462 | 0.0924*** | 0.0954*** |
|  | (1.162) | (0.0394) | (1.835) | (1.556) | (0.0461) | (0.0332) | (0.0310) |
| Profound disability | 3.628*** | 0.105** | 6.378*** | 9.549*** | -0.0803 | -0.126*** | -0.0300 |
|  | (0.989) | (0.0462) | (1.988) | (1.977) | (0.0598) | (0.0419) | (0.0381) |
| Rurality: Inner regional | -2.975 | 0.00448 | 0.170 | -10.27** | 0.135 | 0.137 | 0.0788 |
|  | (2.783) | (0.145) | (3.423) | (4.707) | (0.163) | (0.118) | (0.0735) |
| Rurality: Outer regional and remote | -6.481** | 0.0695 | -10.82* | -22.91*** | 0.152 | 0.288** | 0.151 |
|  | (3.243) | (0.164) | (6.360) | (5.838) | (0.249) | (0.143) | (0.103) |
| Psychosocial disability | 4.523*** | 0.0450 | 7.420*** | 2.579 | -0.0114 | 0.00976 | 0.0415 |
|  | (1.689) | (0.0399) | (2.791) | (1.855) | (0.0507) | (0.0439) | (0.0284) |
| Unemployment rate | -0.491 | 0.0163 | -0.918 | 1.494 | 0.0354 | -0.00430 | 0.00782 |
|  | (0.757) | (0.0246) | (1.663) | (1.201) | (0.0331) | (0.0256) | (0.0213) |
| Constant | 2.878 | 0.578** | -0.976 | 12.32 | -0.570 | 0.746*** | 0.875*** |
|  | (9.059) | (0.252) | (16.96) | (9.749) | (0.357) | (0.243) | (0.191) |
| Observations | 1,535 | 1,535 | 694 | 1,535 | 943 | 1,535 | 1,535 |
| R-squared | 0.083 | 0.093 | 0.127 | 0.126 | 0.164 | 0.071 | 0.099 |
| Number of LGAs | 213 | 213 | 161 | 213 | 186 | 213 | 213 |

Notes: Robust standard errors in parentheses, and they are clustered on the LGA-level; *** p<0.01, ** p<0.05, * p<0.1
