# Supplementary material for: Exploring the impact of a personalised disability reform on people with disability and their primary carers: Evidence from the Australian national disability insurance scheme
Source: PLoS One. 2025 May 7;20(5):e0321377. doi: 10.1371/journal.pone.0321377 (PMC12057950; doi:10.1371/journal.pone.0321377)
Supplement: S14 Table — (DOCX) [file pone.0321377.s014.docx]

### Table S14: Sensitivity Analysis—Margins of Treatment * Post

### (accounting for characteristics interaction with the treatment* post variable)

|  | **(1)** | **(2)** | **(3)** | **(4)** | **(5)** | **(6)** | **(7)** |
| --- | --- | --- | --- | --- | --- | --- | --- |
|  | **Formal services Overall** | **Formal services extensive margin** | **Formal services intensive margin** | **Caring hours** | **Employment** | **Social participation (Alone)** | **Social participation (Any)** |
| NDIS available area # Wave 18 | 0.333 | -0.107 | -0.322 | 4.333 | -0.053 | -0.064 | -0.100 |
|  | (2.471) | (0.0786) | (4.1801) | (3.864) | (0.0862) | (0.0794) | (0.0658) |
| Observations | 1,535 | 1,535 | 694 | 1,535 | 943 | 1,535 | 1,535 |
| R-squared | 0.083 | 0.093 | 0.127 | 0.126 | 0.164 | 0.071 | 0.099 |
| Number of LGAs | 213 | 213 | 161 | 213 | 186 | 213 | 213 |

Notes: Robust standard errors in parentheses, and they are clustered on the LGA-level; tested for p<0.01, p<0.05, p<0.1, and all the results are statistically insignificant
