# Supplementary material for: Exploring the impact of a personalised disability reform on people with disability and their primary carers: Evidence from the Australian national disability insurance scheme
Source: PLoS One. 2025 May 7;20(5):e0321377. doi: 10.1371/journal.pone.0321377 (PMC12057950; doi:10.1371/journal.pone.0321377)
Supplement: S15 Table — (DOCX) [file pone.0321377.s015.docx]

### Table S15: Placebo analysis on primary carers of non-NDIS-eligible caring recipients

|  | **(1)** | **(2)** | **(3)** | **(4)** | **(5)** | **(6)** | **(7)** |
| --- | --- | --- | --- | --- | --- | --- | --- |
|  | **Formal services Overall** | **Formal services extensive margin** | **Formal services intensive margin** | **Caring hours** | **Employment** | **Social participation (Alone)** | **Social participation (Any)** |
| NDIS available area # Wave 18 | 0.466 | 0.466 | -0.152 | -5.438 | 0.0780 | -0.0207 | -0.0550 |
|  | (1.180) | (1.180) | (3.192) | (3.791) | (0.151) | (0.0768) | (0.0721) |
| Wave 18 | -1.622** | -1.622** | -3.303 | 3.344 | -0.0923 | 0.0200 | 0.0472 |
|  | (0.806) | (0.806) | (2.613) | (3.285) | (0.133) | (0.0662) | (0.0636) |
| **Carer Characteristics** |  |  |  |  |  |  |  |
| Age of carer | 0.112 | 0.112 | 0.118 | 0.504 | 0.0216 | 0.0179** | 0.00882 |
|  | (0.254) | (0.254) | (0.535) | (0.372) | (0.0226) | (0.00779) | (0.00667) |
| Age square of carer | -0.00112 | -0.00112 | -0.00209 | -0.00502 | -0.000237 | -0.000172** | -8.19e-05 |
|  | (0.00207) | (0.00207) | (0.00423) | (0.00304) | (0.000237) | (6.68e-05) | (5.78e-05) |
| Number of recipients of care | -0.194 | -0.194 | 0.0424 | 6.128*** | -0.0338 | 0.00271 | -0.0167 |
|  | (0.358) | (0.358) | (0.938) | (1.569) | (0.0494) | (0.0349) | (0.0291) |
| Adults (>=15yo) without disability | -0.743* | -0.743* | -1.231 | -0.778 | 0.0683* | 0.0269 | -0.0117 |
|  | (0.433) | (0.433) | (1.108) | (1.000) | (0.0397) | (0.0201) | (0.0209) |
| Male | 0.721 | 0.721 | 1.844 | -2.531 | -0.0796 | -0.0100 | -0.0355 |
|  | (0.892) | (0.892) | (1.924) | (2.018) | (0.0760) | (0.0444) | (0.0426) |
| Highest education: Bachelor and above | -0.184 | -0.184 | -4.440* | -6.358*** | 0.478*** | 0.125* | 0.123*** |
|  | (0.903) | (0.903) | (2.561) | (2.186) | (0.0750) | (0.0669) | (0.0445) |
| Highest education: Certificates/diploma | -0.557 | -0.557 | -3.004 | -2.386 | 0.283*** | 0.149*** | 0.0735* |
|  | (0.676) | (0.676) | (1.961) | (2.044) | (0.0825) | (0.0460) | (0.0388) |
| Highest education: Year 12 | -0.479 | -0.479 | -1.676 | 0.102 | 0.413*** | 0.0807 | 0.0971** |
|  | (0.916) | (0.916) | (1.873) | (1.834) | (0.0870) | (0.0553) | (0.0434) |
| **Recipient Characteristics** |  |  |  |  |  |  |  |
| Age | -0.150* | -0.150* | 0.0216 | -0.579** | 0.00297 | -0.00416 | -0.00577 |
|  | (0.0834) | (0.0834) | (0.159) | (0.239) | (0.00725) | (0.00494) | (0.00432) |
| Age square | 0.00145** | 0.00145** | 0.000571 | 0.00720*** | -1.73e-05 | 3.75e-05 | 3.10e-05 |
|  | (0.000708) | (0.000708) | (0.00129) | (0.00193) | (6.60e-05) | (4.36e-05) | (4.04e-05) |
| Number of bedrooms | 0.0524 | 0.0524 | 0.871 | -1.673* | -0.000651 | 0.0281 | 0.0505*** |
|  | (0.283) | (0.283) | (0.902) | (1.006) | (0.0385) | (0.0221) | (0.0189) |
| Male | 0.218 | 0.218 | 0.846 | -0.671 | -0.0307 | -0.00487 | -0.0612 |
|  | (0.702) | (0.702) | (1.521) | (2.023) | (0.0582) | (0.0485) | (0.0419) |
| Married/De facto | -1.024 | -1.024 | -2.502 | -0.295 | -0.000129 | 0.0683 | 0.0703 |
|  | (0.744) | (0.744) | (2.053) | (2.726) | (0.0856) | (0.0606) | (0.0599) |
| Highest education: Bachelor and above | -0.646 | -0.646 | -2.252 | -1.722 | 0.134 | 0.0886 | 0.0964** |
|  | (0.799) | (0.799) | (1.694) | (2.967) | (0.0953) | (0.0585) | (0.0434) |
| Highest education: Certificates/diploma | -0.311 | -0.311 | -3.702* | 1.790 | -0.00336 | 0.0418 | 0.0985*** |
|  | (0.682) | (0.682) | (1.997) | (1.732) | (0.0970) | (0.0386) | (0.0308) |
| Highest education: Year 12 | -0.770 | -0.770 | -2.885 | 1.570 | -0.00360 | -0.0183 | 0.0127 |
|  | (0.795) | (0.795) | (2.121) | (2.650) | (0.130) | (0.0695) | (0.0632) |
| Born in Australia mainland | -0.305 | -0.305 | -2.181 | -1.962 | 0.0961 | 0.101*** | 0.0490 |
|  | (0.755) | (0.755) | (2.208) | (1.939) | (0.0800) | (0.0382) | (0.0440) |
| Profound disability | 1.429*** | 1.429*** | 4.730*** | 7.921*** | -0.0449 | -0.0931* | -0.0564 |
|  | (0.444) | (0.444) | (1.759) | (2.618) | (0.112) | (0.0557) | (0.0508) |
| Rurality: Inner regional | -1.358 | -1.358 | -3.017 | -7.151 | 0.162 | 0.0791 | 0.148** |
|  | (1.028) | (1.028) | (2.260) | (6.594) | (0.140) | (0.0997) | (0.0621) |
| Rurality: Outer regional and remote | -1.638 | -1.638 | -6.013 | -10.86 | 0.267 | 0.202 | 0.210** |
|  | (1.913) | (1.913) | (4.813) | (6.592) | (0.200) | (0.149) | (0.0902) |
| Psychosocial disability | -0.343 | -0.343 | 3.236 | -0.393 | 0.275*** | 0.0291 | 0.0312 |
|  | (0.945) | (0.945) | (2.526) | (2.539) | (0.0947) | (0.0611) | (0.0535) |
| Unemployment rate | 0.128 | 0.128 | 0.116 | 0.207 | -0.0526 | 0.0443 | 0.0567** |
|  | (0.332) | (0.332) | (0.944) | (1.640) | (0.0632) | (0.0300) | (0.0269) |
| Constant | 3.134 | 3.134 | 2.624 | 21.11 | -0.171 | -0.276 | 0.158 |
|  | (7.023) | (7.023) | (13.34) | (16.57) | (0.688) | (0.327) | (0.272) |
| Observations | 1,066 | 1,066 | 456 | 1,066 | 392 | 1,066 | 1,066 |
| R-squared | 0.025 | 0.025 | 0.085 | 0.092 | 0.229 | 0.068 | 0.069 |
| Number of LGAs | 213 | 213 | 149 | 213 | 147 | 213 | 213 |

Notes: Robust standard errors in parentheses, and they are clustered on the LGA level; *** p<0.01, ** p<0.05, * p<0.1
