# Supplementary material for: Exploring the impact of a personalised disability reform on people with disability and their primary carers: Evidence from the Australian national disability insurance scheme
Source: PLoS One. 2025 May 7;20(5):e0321377. doi: 10.1371/journal.pone.0321377 (PMC12057950; doi:10.1371/journal.pone.0321377)
Supplement: S1 Fig — (DOCX) [file pone.0321377.s016.docx]

### Fig S1: The flowchart of sample restriction and sample size
