# Supplementary material for: Exploring the impact of a personalised disability reform on people with disability and their primary carers: Evidence from the Australian national disability insurance scheme
Source: PLoS One. 2025 May 7;20(5):e0321377. doi: 10.1371/journal.pone.0321377 (PMC12057950; doi:10.1371/journal.pone.0321377)

### Fig S2a-2e: Results of sample size estimation by outcomes of interest

**Fig S2a. Formal services overall**


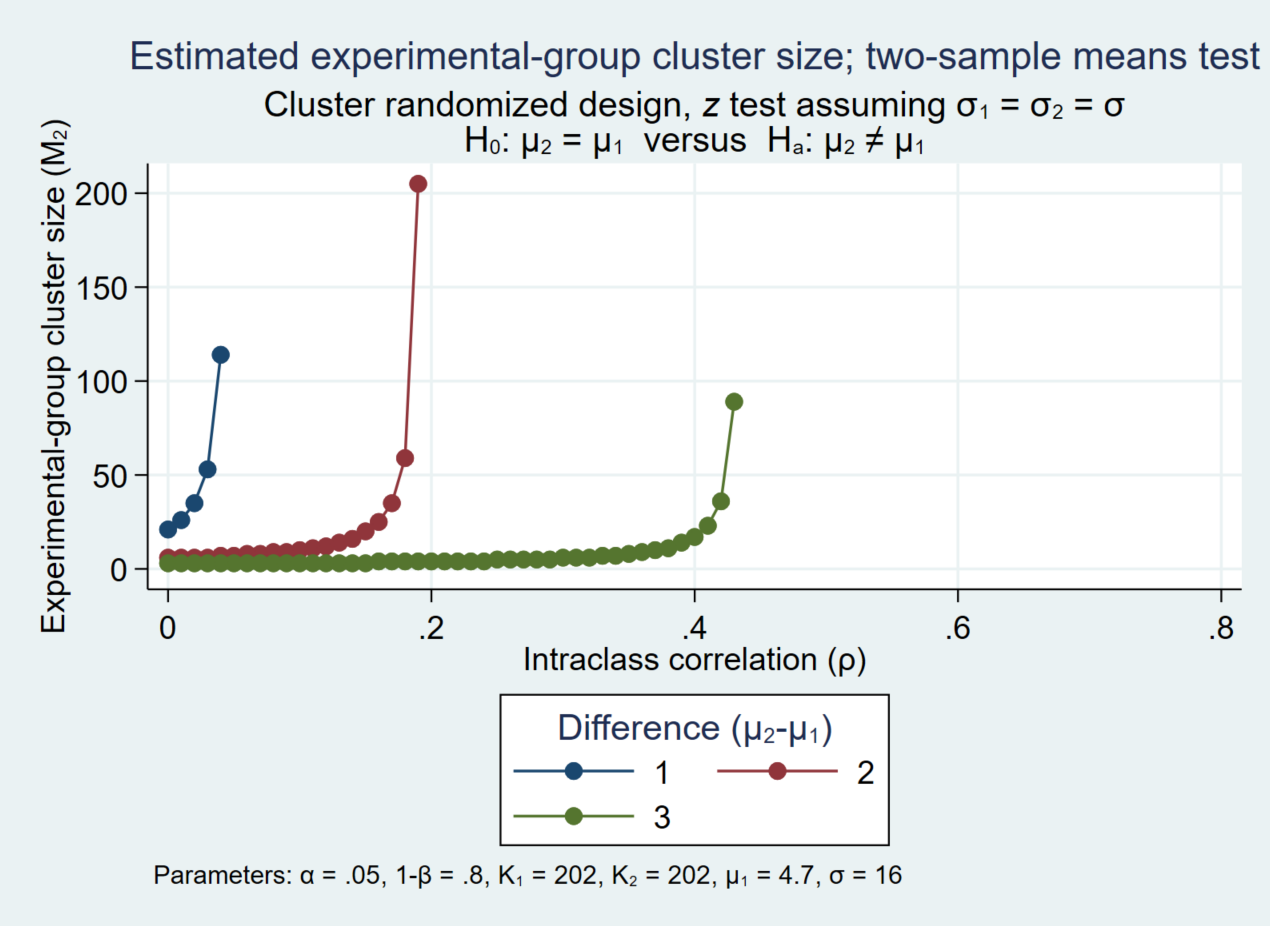


**Fig S2b: Caring hours(times/week)**


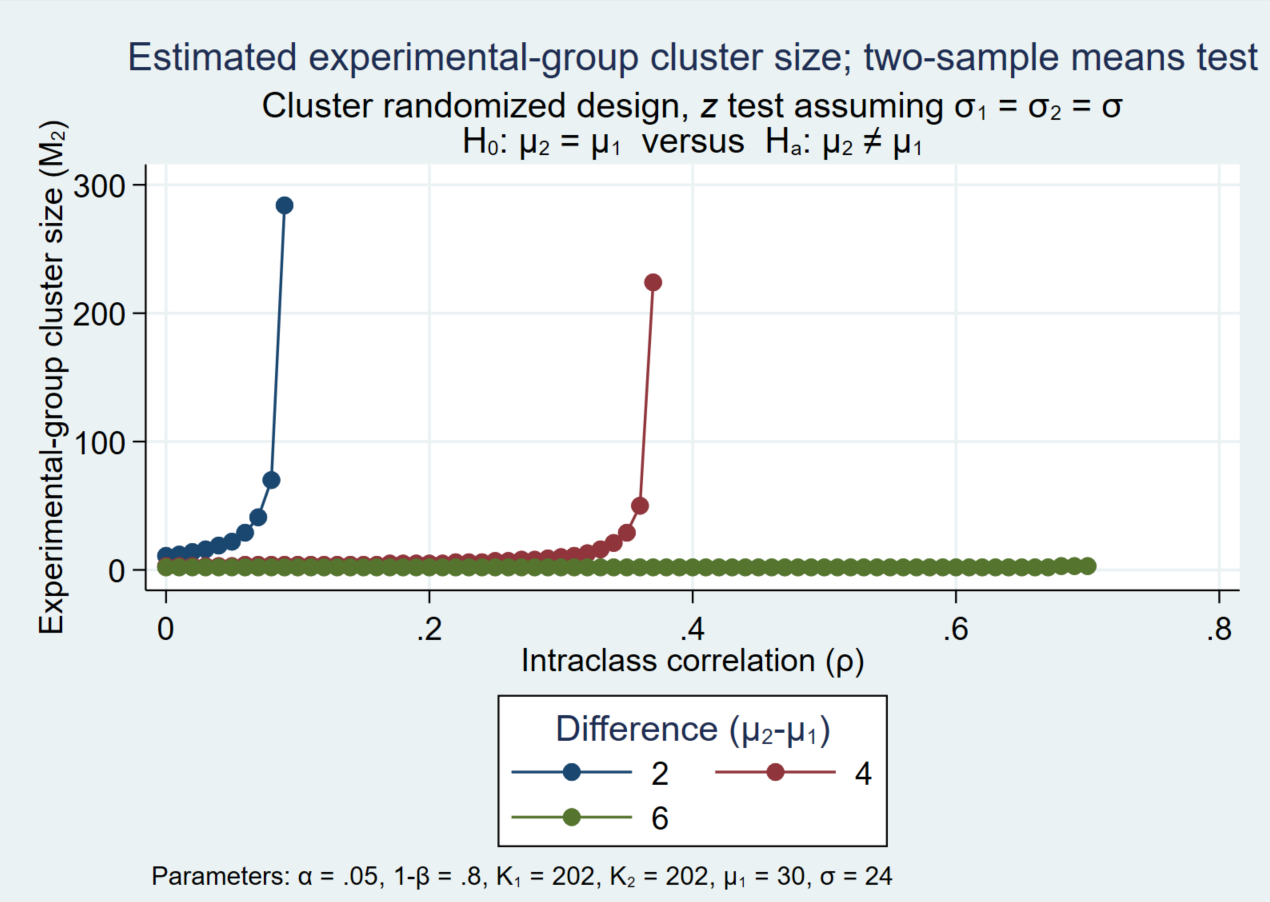


**Fig S2b-1: Caring hours zoom in(times/week) with a difference of 6**


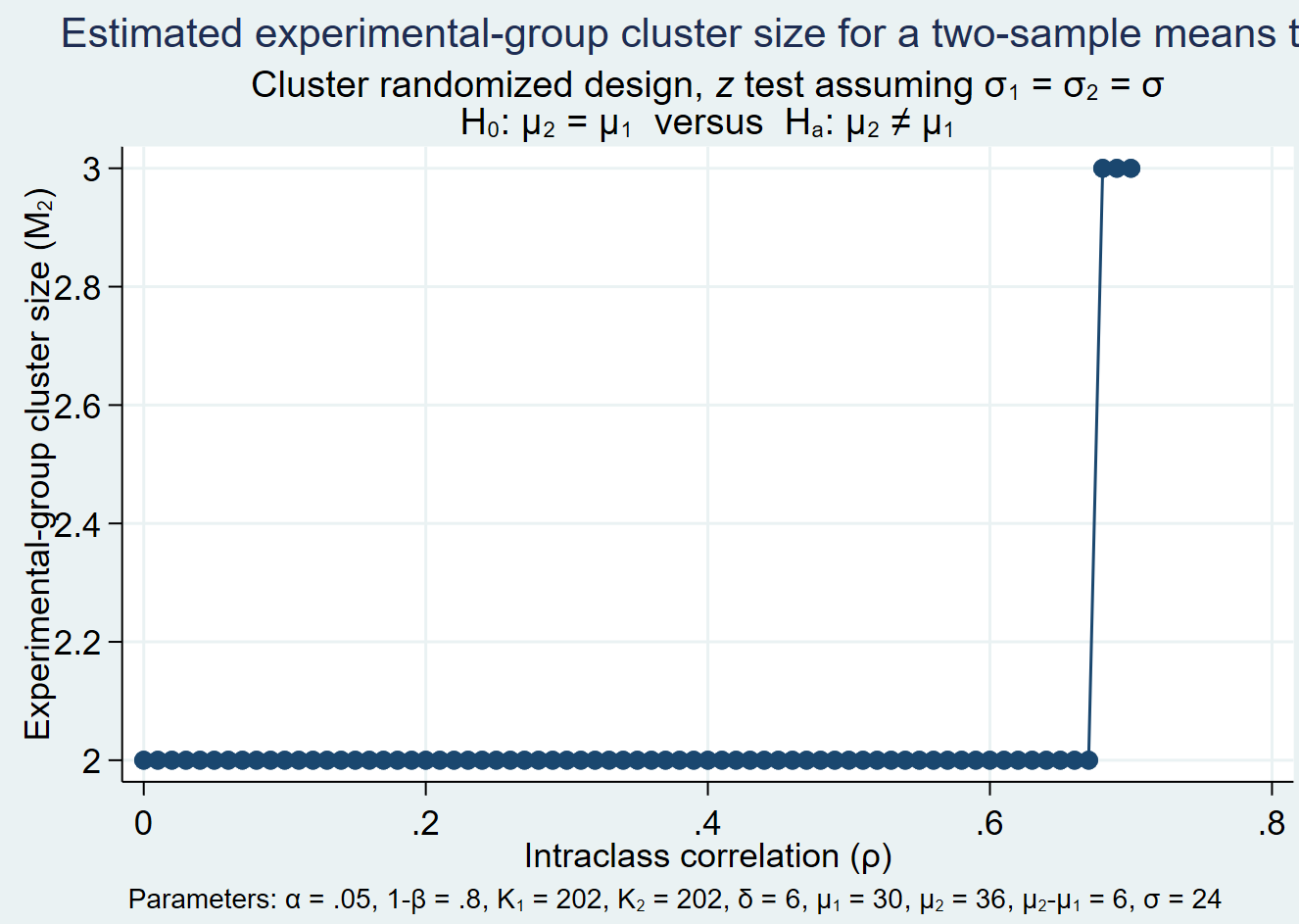


**Fig S2c: Employment**


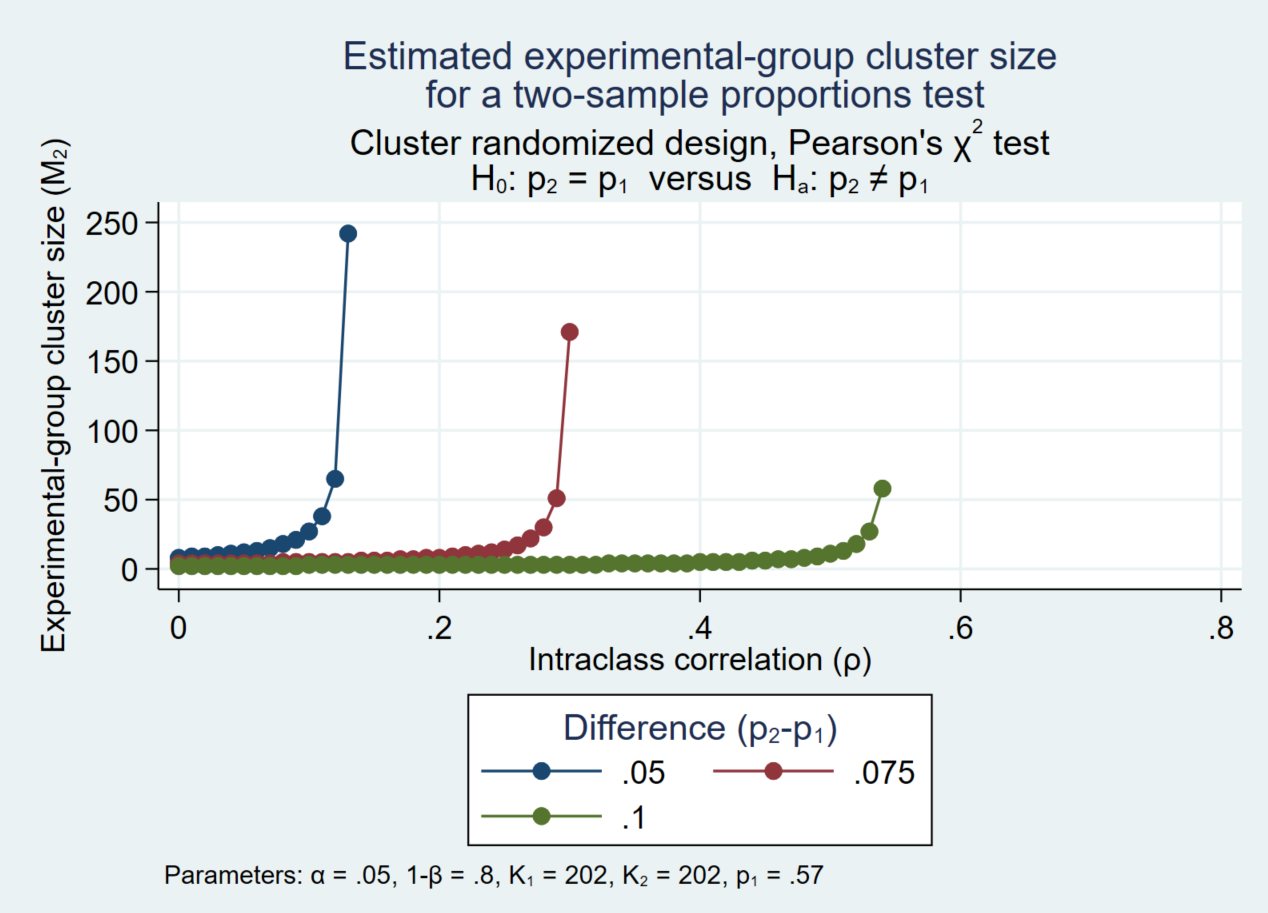


**Fig S2c-1: Employment zoom in (Left with a difference of 0.075 AND right of 0.1)**


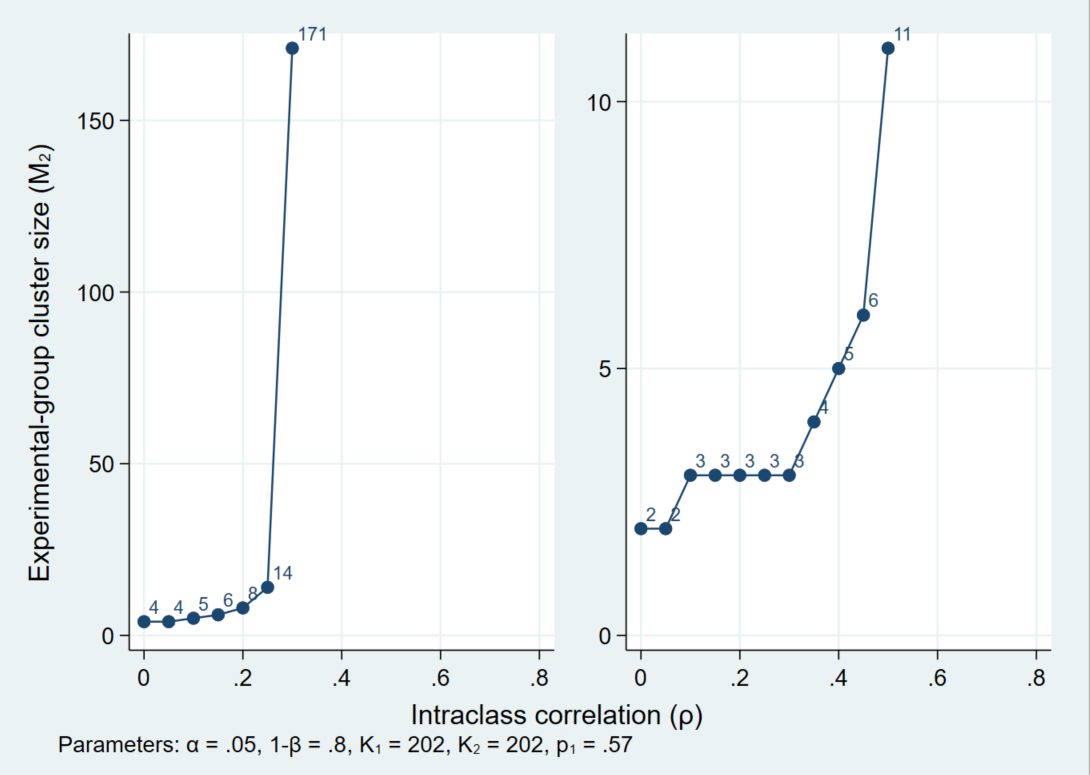


**Fig S2d: Social Participation (Alone)**


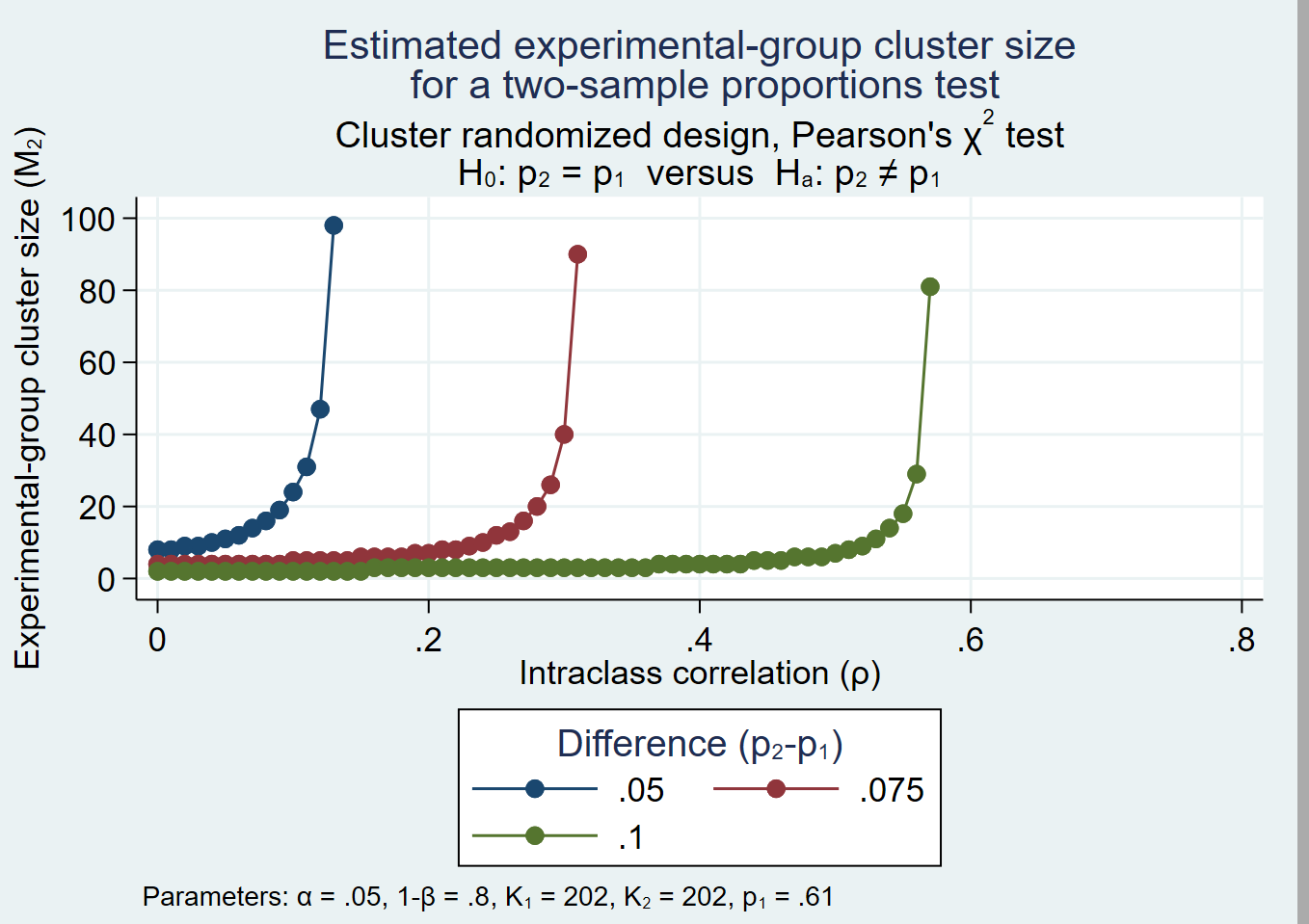


**Fig S2e: Social participation (Any)**


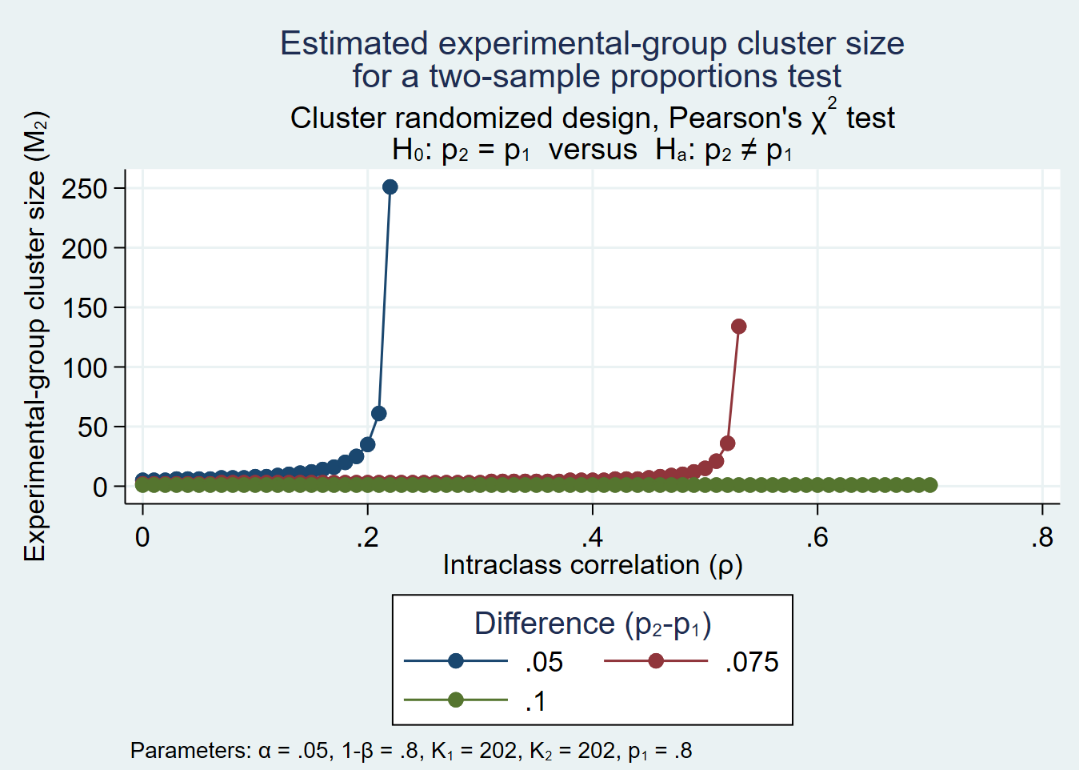

Supplement: S2a-S2e Fig — (DOCX) [file pone.0321377.s017.docx]
